# A Novel Protein, CHRONO, Functions as a Core Component of the Mammalian Circadian Clock

**DOI:** 10.1371/journal.pbio.1001839

**Published:** 2014-04-15

**Authors:** Akihiro Goriki, Fumiyuki Hatanaka, Jihwan Myung, Jae Kyoung Kim, Takashi Yoritaka, Shintaro Tanoue, Takaya Abe, Hiroshi Kiyonari, Katsumi Fujimoto, Yukio Kato, Takashi Todo, Akio Matsubara, Daniel Forger, Toru Takumi

**Affiliations:** 1 RIKEN Brain Science Institute, Wako, Saitama, Japan; 2 Graduate School of Biomedical Sciences, Hiroshima University, Minami, Hiroshima, Japan; 3 Department of Mathematics, University of Michigan, Ann Arbor, Michigan, United States of America; 4 Laboratory for Animal Resources and Genetic Engineering, RIKEN Center for Developmental Biology, Chuo, Kobe, Japan; 5 Department of Radiation Biology and Medical Genetics, Graduate School of Medicine, Osaka University, Suita, Osaka, Japan; 6 Core Research for Evolutional Science and Technology, Japan Science and Technology Agency, Chiyoda, Tokyo, Japan; University of Geneva, Switzerland

## Abstract

Circadian rhythms are controlled by a system of negative and positive genetic feedback loops composed of clock genes. Although many genes have been implicated in these feedback loops, it is unclear whether our current list of clock genes is exhaustive. We have recently identified *Chrono* as a robustly cycling transcript through genome-wide profiling of BMAL1 binding on the E-box. Here, we explore the role of *Chrono* in cellular timekeeping. Remarkably, endogenous CHRONO occupancy around E-boxes shows a circadian oscillation antiphasic to BMAL1. Overexpression of *Chrono* leads to suppression of BMAL1–CLOCK activity in a histone deacetylase (HDAC) –dependent manner. *In vivo* loss-of-function studies of *Chrono* including *Avp* neuron-specific knockout (KO) mice display a longer circadian period of locomotor activity. *Chrono* KO also alters the expression of core clock genes and impairs the response of the circadian clock to stress. CHRONO forms a complex with the glucocorticoid receptor and mediates glucocorticoid response. Our comprehensive study spotlights a previously unrecognized clock component of an unsuspected negative circadian feedback loop that is independent of another negative regulator, *Cry2*, and that integrates behavioral stress and epigenetic control for efficient metabolic integration of the clock.

## Abstract 2

Two independent studies, one of them using a computational approach, identified CHRONO, a gene shown to modulate the activity of circadian transcription factors and alter circadian behavior in mice.

## Author Summary

The circadian clock has a fundamental role in regulating biological temporal rhythms in organisms, and it is tightly controlled by a molecular circuit consisting of positive and negative regulatory feedback loops. Although many of the clock genes comprising this circuit have been identified, there are still some critical components missing. Here, we characterize a circadian gene renamed Chrono (Gm129) and show that it functions as a transcriptional repressor of the negative feedback loop in the mammalian clock. Chrono binds to the regulatory region of clock genes and its occupancy oscillates in a circadian manner. Chrono knockout and Avp-neuron-specific knockout mice display longer circadian periods and altered expression of core clock genes. We show that Chrono-mediated repression involves the suppression of BMAL1–CLOCK activity via an epigenetic mechanism and that it regulates metabolic pathways triggered by behavioral stress. Our study suggests that Chrono functions as a clock repressor and reveals the molecular mechanisms underlying its function.

## Introduction

Circadian rhythms with a period of approximately 24 h endow organisms with the ability to adapt to changes of solar light following earth's rotation. The mammalian circadian clock system consists of inputs from light and feeding, a core pacemaker located in a paired nuclei, called suprachiasmatic nucleus (SCN), and outputs including, but not limited to, cycles of locomotor activity, sleep–awake, and hormonal secretion. Disturbance of the biological clock causes not only sleep rhythm disruptions but also various pathological conditions such as cancer, metabolic, and psychiatric disorders [1]–[5].

The clock gene, *period*, was first identified in fly [6]–[8] and later in various organisms [9]. The molecular mechanism of circadian transcription was then found to be based on interconnected transcription–translation feedback loops (TTFL), conserved from prokaryotes to humans [10]–[12]. In mammals, the complex of positive elements BMAL–CLOCK (NPAS2) activates PER and CRY that repress their own transcription to form a negative feedback loop. An accessory feedback loop involves ROR and REV–ERBα, which regulate BMAL1 transcription positively and negatively, respectively, whereas BMAL1 activates REV–ERBα expression. More members of the circadian clock components have been identified [13]–[16]. Because of the complexity of circadian timekeeping, mathematical modeling has emerged as an important tool to understand data and make novel predictions [17]–[19]. In particular, a recently published mathematical model reproduces much of the known data on circadian timekeeping (e.g., mutant phenotypes) and correctly predicts the pharmacological manipulation of circadian rhythms [20],[21].

Although it is widely believed that the major components of the mammalian circadian clock have been identified, the search for additional clock components continues. In our previous study [22] and in others [23],[24], the systematic screening by using ChIP (Chromatin immunoprecipitation)–Chip and ChIP-seq revealed several uncharacterized genome-wide BMAL1 targets. Among strong BMAL1 binding sites, only one novel gene, *Gm129*, was found besides known clock and clock-controlled genes such as *Per1*, *Per2*, *Cry1*, *Cry2*, *Dbp*, and *Tef. Gm129* expression shows a robust circadian rhythm antiphasic to *Bmal1*. In light of its circadian expression, *Gm129*, now renamed *Chrono* (ChIP-derived Repressor of Network Oscillator), appeared to be a core clock gene.

Here, we study the functional role of *Chrono* in the circadian clock. CHRONO binds to the promoters of clock genes and functions as a negative regulatory component of the circadian clock. *In vivo* loss-of-function of *Chrono* including an *Avp* neuron-specific knockout (KO) mouse model displays a longer circadian period of locomotor activity. We demonstrate that *Chrono* is a core-clock component similar to *Cry2*, although its repression mechanism operates through an independent pathway. *In silico* study using a modified Kim–Forger model predicts that the recently identified residual rhythmicity in the *Cry1*, *Cry2* double KO [25], is dependent on *Chrono*. Remarkably, *Chrono* is involved in glucocorticoid receptor (GR)–mediated metabolic physiology. We conclude that *Chrono* is part of the negative feedback loop of the mammalian circadian clock and a potential link between the clock and stress metabolism.

## Results

### ChIP-Seq Identifies a Novel Clock Gene Regulated by BMAL1

**Figure 1:**
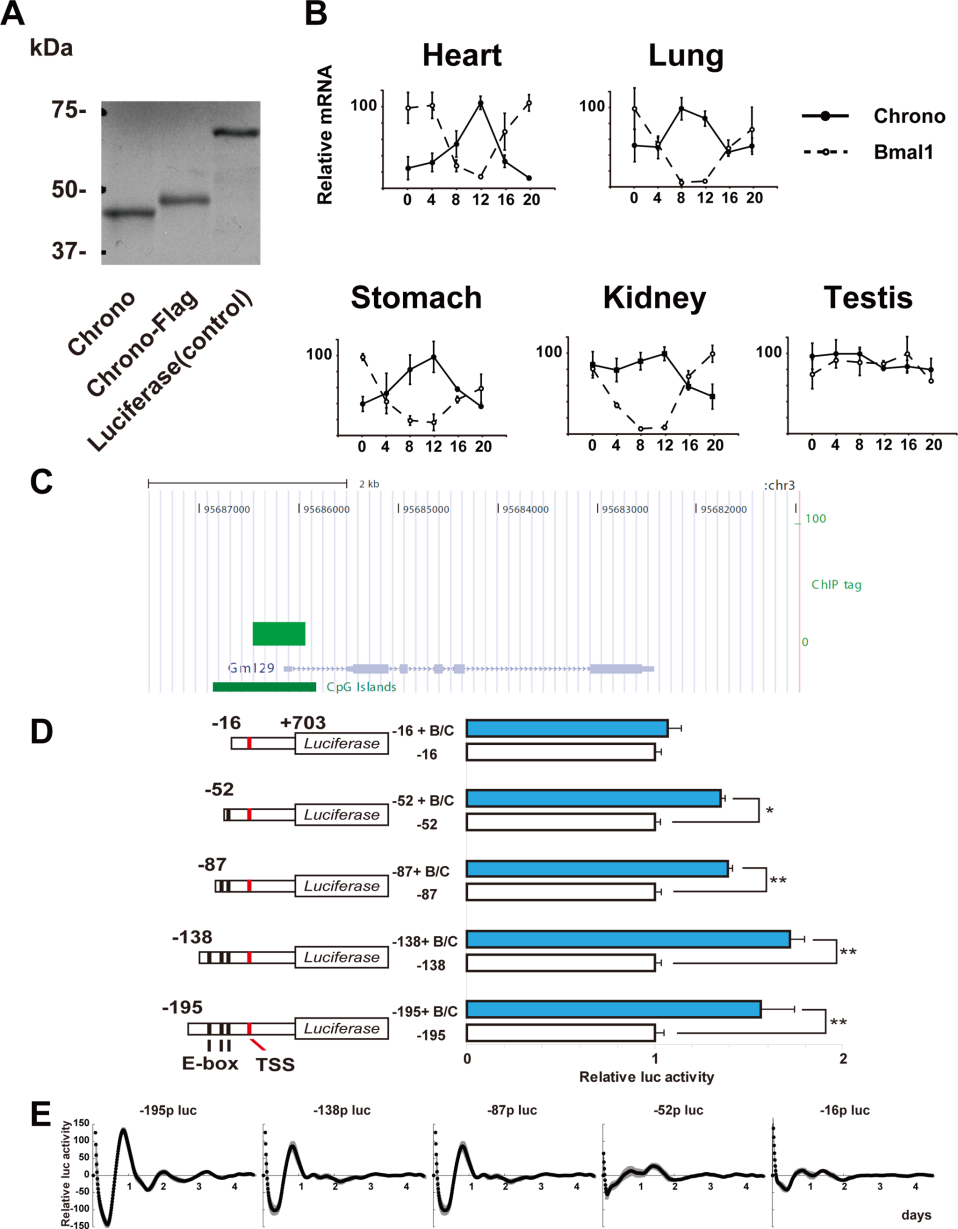
CHRONO. (A) Cell-free synthesis of CHRONO (GM129). Autoradiogram of *in vitro* translated CHRONO, CHRONO–FLAG, and luciferase (control). Protein was synthesized in the presence of [^35^S]methionine and resolved on a 10% SDS-PAGE gel. (B) Temporal mRNA expression of *Chrono* (solid lines with black circles) and *Bmal1* (dotted lines with white circles) in mouse peripheral tissues. The abscissa represents time in CT and the ordinate the mRNA amounts. The relative levels of mRNA were normalized to the corresponding glyceraldehyde-3-phosphate dehydrogenase (GAPDH) mRNA levels. The maximum mRNA amount was set to 100. Plots and error bars represent mean ± S.E.M of triplicate samples. (C) BMAL1 ChIP-seq tag enrichments in the whole brain sample were located on the *Chrono* promoter region in the UCSC genome browser view. (D) The effects of overexpression of BMAL1 and CLOCK (B/C) on the *Chrono* promoter modification were evaluated by using a luciferase assay. The left scheme shows each construct on a black line indicating the position of the E-box element and a red line indicating the TSS. The basal transcriptional activity of each *Chrono* promoter was set to 1. Horizontal bars represent means ± S.E.M. of four samples (*p<0.005, **p<0.0005, Student's *t* test). (E) E-boxes of the *Chrono* promoter required for transcriptional oscillation. The cell-culture-based bioluminescent reporter assay was performed with the each construct in (D). The abscissa indicates the day in culture, and the ordinate the relative bioluminescence intensity (kcpm, 1,000 photon counts per minute). Shaded area indicates ± S.E.M of four samples.

Our previous ChIP-based genome-wide analyses using a core clock transcription factor BMAL1 identified hundreds of target molecules [22]. Among these targets, *Gm129*, now called *Chrono*, was one of the groups with the strongest binding including core clock proteins PER and CRY. Another ChIP-seq experiment using *in vivo* brain samples also identified *Chrono* as a BMAL1 target. *Chrono* exists only in mammals, is well conserved among mammals (Figure S1), and consists of 375 amino acids with no functional domains. To examine whether *Chrono* encodes a polypeptide, we performed an *in vitro* translation experiment. Bands of approximately 45 kDa (CHRONO) and 46 kDa (CHRONO–FLAG) were observed as an *in vitro* translation product (Figure 1A).

### *Chrono* Transcripts Display Robust Circadian Expression

Our previous study showed robust circadian oscillation of *Chrono* mRNA in the mouse SCN and liver [22]. We further examined its expression in five different mouse peripheral tissues (heart, lung, stomach, kidney, and testis) by quantitative RT-PCR. After entrainment of mice housed for 2 wk under a 12–12 h light–dark (LD) cycle, samples were collected every 4 h starting at circadian time (CT) 0 (*n* = 3 at each time point) in the third dark–dark (DD) cycle. The temporal expression of *Chrono* transcripts in all tested tissues except testis displayed robust circadian rhythms peaking at approximately CT 12 (Figure 1B), which were antiphasic to *Bmal1*. This result supports that *Chrono* encodes a component of the circadian clock loops [26]. The ChIP-seq experiment using brain samples revealed that BMAL1 strongly binds to CpG islands on the *Chrono* promoter *in vivo* (Figure 1C). Studying differently sized *Chrono* promoter constructs (−195, −138, −87, −52, and −16 bp from the transcriptional start site (TSS) of *Chrono*/PGL3B) showed that the closest E-box to the TSS is necessary to generate circadian oscillation of *Chrono* in NIH3T3 cells and that all of the three E-boxes contribute to robust circadian rhythms (Figure 1D and E). These results suggest that BMAL1 strongly binds to the E-boxes on the *Chrono* promoter and regulates circadian expression of *Chrono*, making *Chrono* a novel clock gene.

We next asked whether CHRONO is also expressed rhythmically at the protein level. We prepared liver samples at CT 2, 8, 14, and 20. We raised a specific antibody against the CHRONO protein. CHRONO showed circadian rhythm antiphasic to BMAL1 as in the mRNA expression (Figure S2A and C). This oscillation was observed in both the mouse CHRONO antibody we generated and the human C1orf51 antibody (ab106120, Abcam) (Figure S2B).

### CHRONO Forms a Complex with Other Clock Components

**Figure 2:**
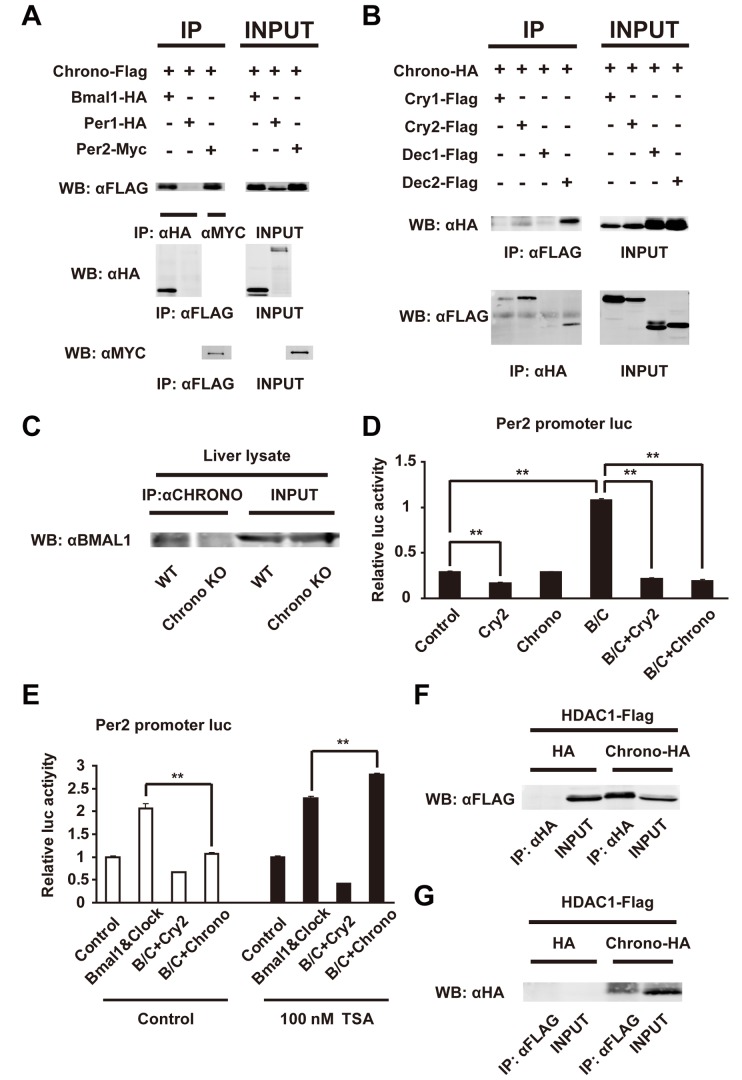
CHRONO's interaction and the role of CHRONO as a transcriptional repressor. (A and B) Immunoblots show expression of proteins in COS7 cells transfected with the indicated plasmids after IP with antibodies. INPUT indicates immunoblotting results of total cell lysates. (C) Endogenous BMAL1 and CHRONO interaction in the WT and *Chrono* KO mice liver. (D) Effects of *Chrono* expression on the *Per2* promoter luciferase activity. Overexpressed CHRONO repressed transactivation by exogenous expression of BMAL1 and CLOCK (B/C) in the *Per2* promoter in a similar way to *Cry2*. Bars represent means ± S.E.M. of four samples (**p<0.001, Student's *t* test). (E) An HDAC inhibitor (100 nM TSA) restored the *Chrono*-derived repression of transactivation with BMAL1 and CLOCK (B/C) on the *Per2* promoter. The basal transcriptional activity of *Per2* promoter in each experiment was set to 1. Data are means ± S.E.M. of four samples (**p<0.001, Student's *t* test). (F and G) CHRONO and HDAC1 interaction *in vitro*. Co-IP assays of COS7 cells using HA-tagged CHRONO and FLAG-tagged HDAC1 as indicated. HA protein was used as negative control.

Because CHRONO showed a similar rhythmic expression profile to other core clock proteins, we asked if CHRONO binds directly with clock proteins. Various clock proteins with tags were expressed in COS7 cells and the expression was assessed by immunoprecipitation (IP) and blotting with anti-tag antibodies. CHRONO bound to BMAL1, PER2, CRY2, and DEC2 but not to PER1, CRY1, and DEC1 (Figure 2A and B and Figure S2D). Among these interactions, we asked if CHRONO–BMAL1 binding occurs endogenously *in vivo*. We observed a band of BMAL1 in the CHRONO antibody IP from mouse liver lysate that was absent in the IP from *Chrono*-deficient mouse liver (Figure 2C). These results suggest that CHRONO endogenously binds to BMAL1 *in vivo*.

### *Chrono* Is Involved in HDAC-Dependent Transcriptional Repression

Next, we asked how *Chrono* is involved in circadian transcription. The luciferase activity of the *Per2* promoter (∼−2,817+110 bp from TSS/PGL3B) in NIH3T3 cells was repressed by co-expression with *Cry2* and *Dec2* (Figures 2D and S3A). The basic transcription activity of *Per2* was increased by *Bmal1* and *Clock* co-expression, and this activation was repressed by *Chrono* as well as *Cry2*. This repression was also seen in the *Chrono* promoter (−1,333 bp from TSS/PGL3B) (Figure S3B). Moreover, overexpression of *Chrono* reduced the transcriptional amplitude of expression on the *Dbp* promoter, just as *Cry2* (Figure S3C and D). These results suggest that *Chrono* functions as a negative element of circadian transcription, similar to *Cry2*.

Histone modification by histone deacetylase (HDAC) is one of the mechanisms of transcriptional regulation [27]. HDAC is often recruited during the transcriptional repression process. We hypothesized that CHRONO is in a repressor complex that includes CRY2. To investigate the potential role of HDAC in *Chrono*-mediated transcriptional repression, we treated cells with trichostatin A (TSA), an HDAC inhibitor, in the reporter assay. *Chrono*, as well as *Cry2*, repressed the enhanced luciferase activity of the *Per2* promoter. However, in the presence of TSA, *Chrono* did not repress activity but rather enhanced activity, whereas *Cry2* did not change its repression (Figure 2E). To confirm the involvement of HDAC in *Chrono*-mediated transcriptional repression, we investigated the interaction of CHRONO with HDAC1. We expressed and showed co-IP of CHRONO–HA and HDAC1–FLAG in COS7 cells, indicating that CHRONO is bound with HDAC1 (Figure 2F and G).

### CHRONO Rhythmically Binds the E-Boxes on Circadian Gene Promoters

**Figure 3:**
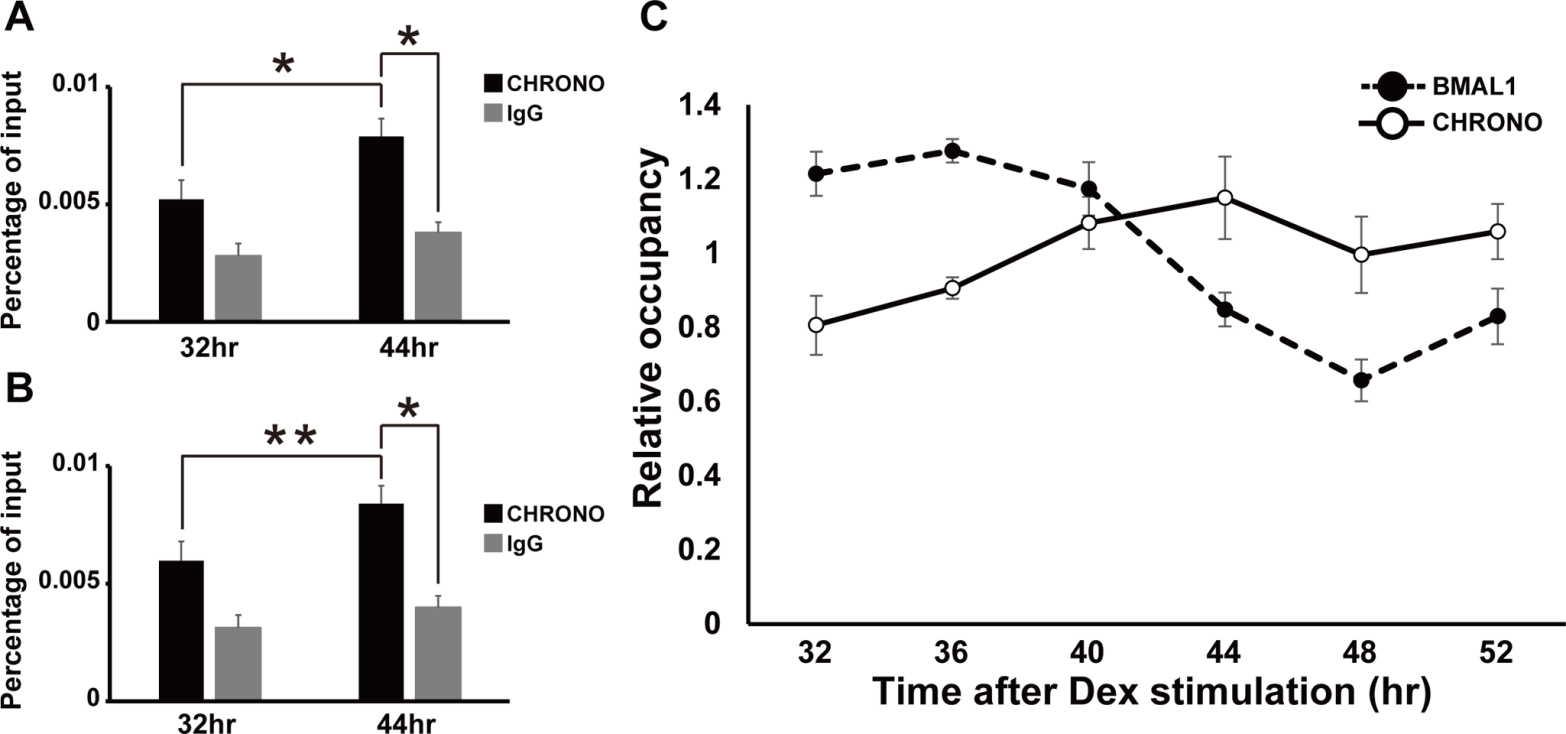
Endogenous CHRONO rhythmically binds to the E-box on circadian gene promoter. (A and B) ChIP analysis for CHRONO and IgG (negative control). The CHRONO occupancies at the endogenous E-boxes of *Per2* (A) and *Dbp* (B) promoters were detected in the two different NIH3T3 cells 32 or 44 h after induction with dexamethasone. The graphs showed relative real-time PCR values compared to input. Data are means ± S.E.M. of three samples (*p<0.05, **p<0.01, Student's *t* test). (C) ChIP abundance of CHRONO and BMAL1 at the *Per2* promoter E-box in NIH3T3 cells after 100 nM dexamethasone stimulation. The graph showed relative real-time PCR values. The data were plotted as percentages relative to the average (1.0). Data are means ± S.E.M. of 3–4 samples.

We then asked if endogenous CHRONO participates in clock function as a core clock component. A ChIP experiment with the CHRONO antibody in NIH3T3 cells after induction with dexamethasone showed endogenous binding of CHRONO to the E-boxes on *Per2* (Figure 3A) and *Dbp* (Figure 3B) promoters. The levels of chromatin occupancy around the E-boxes on *Per2* and *Dbp* were significantly different between 32 h and 44 h after dexamethasone stimulation (Figure 3A and B, *p<0.05, **p<0.01). Moreover, endogenous CHRONO occupancy showed circadian oscillation antiphasic to BMAL1 (Figures 3C and S3G). These results strongly suggest that *Chrono* behaves as an auto-regulated clock component.

### *Chrono* KO Mice Have a Lengthened Circadian Period

**Figure 4:**
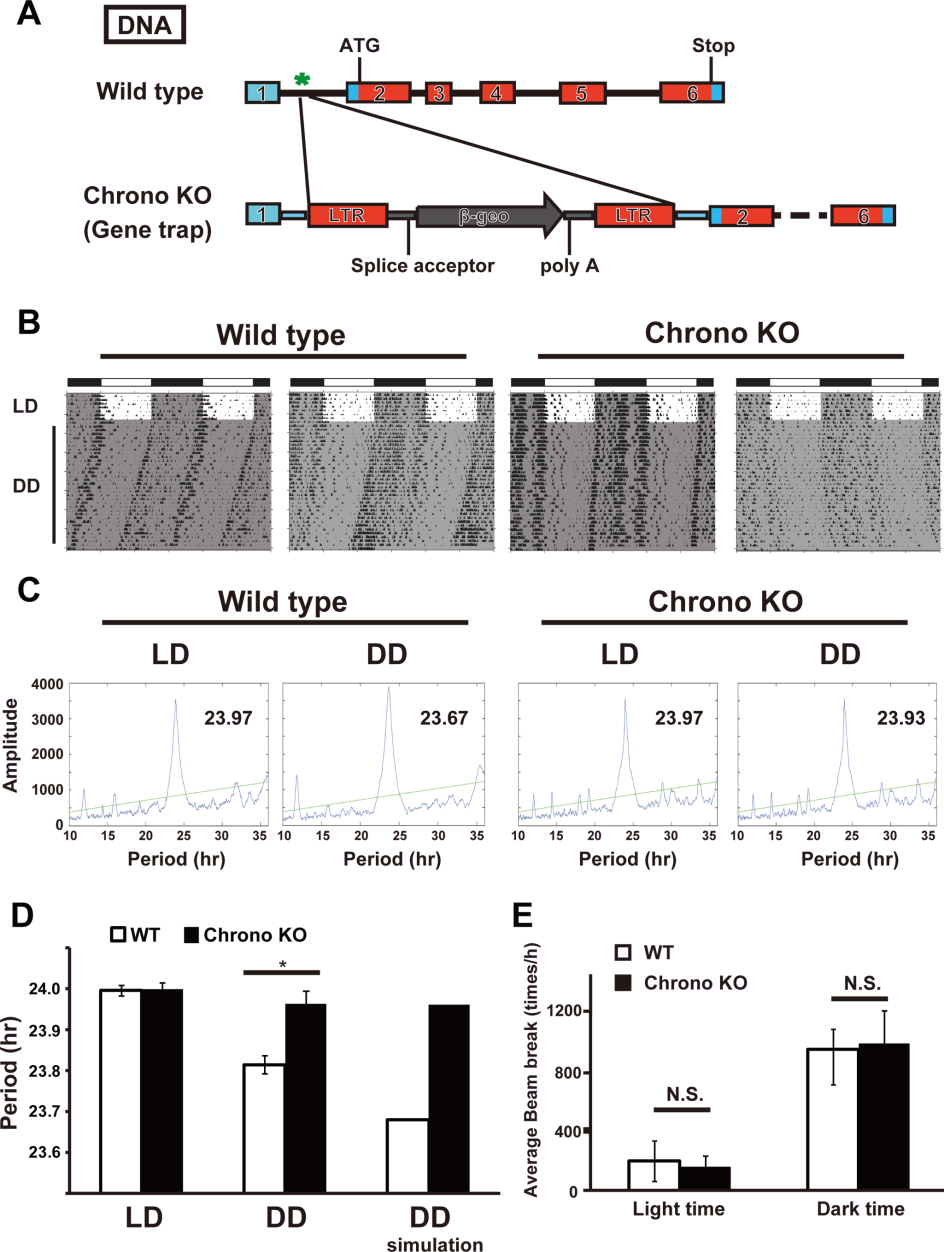
Circadian phenotypes of *Chrono* KO mice. (A) Genomic structure of a portion of the mouse *Chrono* gene. Exons are indicated by heavy lines. Gene trapped (KO) mice expressed different transcripts and translated products from WT mice. (B) Representative actograms of WT and *Chrono* KO mice. Animals were maintained under 12–12 h LD cycles initially and transferred to DD as indicated. Shaded area indicates dark phase. (C) Representative chi-squared periodograms of WT and *Chrono* KO locomotor activities for 1 wk at LD and DD. (D) Comparison of free-running period estimated for WT and *Chrono* KO littermates. Pairwise comparisons found no significant differences of circadian periods between WT and KO under LD (left). However, a significantly longer free-running period was observed in *Chrono* KO mice compared to WT under DD (middle). The computer simulation based on our biochemical data reproduces these phenotypes under DD (right). Free-running periods under DD are 23.96±0.11 h for *Chrono* KO mice (*n* = 12, male) and 23.81±0.08 h for WT siblings (*n* = 13, male) (mean period ± S.D.; *p<0.001, Student's *t* test). (E) Basal locomotor activity. Amounts of activities during light phase (left) and dark phase (right) for 1 wk in LD show no significant differences. *N* = 13 for WT and *n* = 12 for *Chrono* KO.

To evaluate the physiological and circadian clock function of *Chrono in vivo*, we generated *Chrono* KO mice by using a gene trap method (Figures 4A and S4). After entrainment in the 12–12 h LD condition, the locomotor activity rhythm under DD was recorded. In DD the average circadian period length of wild-type (WT) mice was 23.81±0.08 h (mean ± standard deviation, *n* = 13), whereas that of *Chrono*-deficient mice was significantly longer (23.96±0.11 h, *n* = 12) (*p<0.001; Student's *t* test) (Figure 4B–D). To logically confirm that the behavioral *Chrono* KO phenotype is an outcome of the observed biochemical and in vitro characteristics of *Chrono* (Figure 2), we adopt a mathematical modeling approach. We used a recently developed mathematical model of mammalian circadian clock (Kim–Forger model) because this model successfully reproduced and predicted the circadian period change in response to mutations of clock genes (e.g., *Per1/2*, *Cry1/2*, *Bmal1*, *Clock*, and etc.) and pharmacological inhibition of kinase (e.g., CK1δ/ε) [20],[21]. The model is extended to include biochemical mechanisms of *Chrono*, such as binding with other clock components (Figure 2A and B), transcriptional repression by *Chrono* (Figure 2D–G), and rhythms of *Chrono* (Figure 1B) (see details in Text S1). Although CHRONO acts similarly to CRY2, we also incorporate key differences, including their very different mRNA time profiles (Figure S5) and the fact that CHRONO does not bind PER1. When the transcription of *Chrono* is inhibited in the model, the model predicts that *Chrono* KO lengthens the period (Figure 4D, right), which indicates that the biochemical mechanisms we have identified for *Chrono*-mediated repression of BMAL1–CLOCK (Figure 2D) are expected to cause the *Chrono* KO phenotypes. There was no difference of basal locomotor activity between WT and *Chrono* KO mice during the light phase and dark phase after 1 wk in LD (*p*>0.5; Student's *t* test) (Figure 4E). We also examined how *Chrono* KO mice responded to shifts in the LD cycle. When the lighting cycle was advanced 6 h, both WT and *Chrono* KO mice re-entrained progressively over 10 d, and there were no difference between the genotypes (Figure S6A and B). Moreover, no induction of *Chrono* mRNA in the SCN was observed after light stimulation for 30 min (Figure S6C).

### *Chrono* Acts as a Transcriptional Repression Independent of *Cry2*

Because *Chrono* is a putative repressor in the circadian clock mechanism, we next asked whether the expression of clock genes is altered after the deletion of *Chrono* in mice. In mouse embryonic fibroblasts (MEFs) derived from *Chrono* KO mice (Figure S7A), *Per2* expression was increased at 48 h and 52 h after dexamethasone stimulation (Figure S7B), compared with WT MEFs. In the liver derived from *Chrono* KO mice (Figure S7C), *Per3*, *Cry1*, *Dbp*, and *Rev*–*erb*s were increased at CT12 (*p<0.05, **p<0.01, Student's *t* test), consistent with the idea that *Chrono* is involved with negative feedback regulation of the core clock component via E-box. The similar trend was observed in the *Chrono*-deficient SCN (Figure S7D). Moreover, Acetyl-Histone H3 occupancy on *Per2* and *Dbp* promoters was enhanced in *Chrono* KO MEFs compared to WT (Figure S7E and F; **p<0.01, Student's *t* test). This result suggests that *Chrono* changed the epigenetic modification of cells with the expression status.

**Figure 5:**
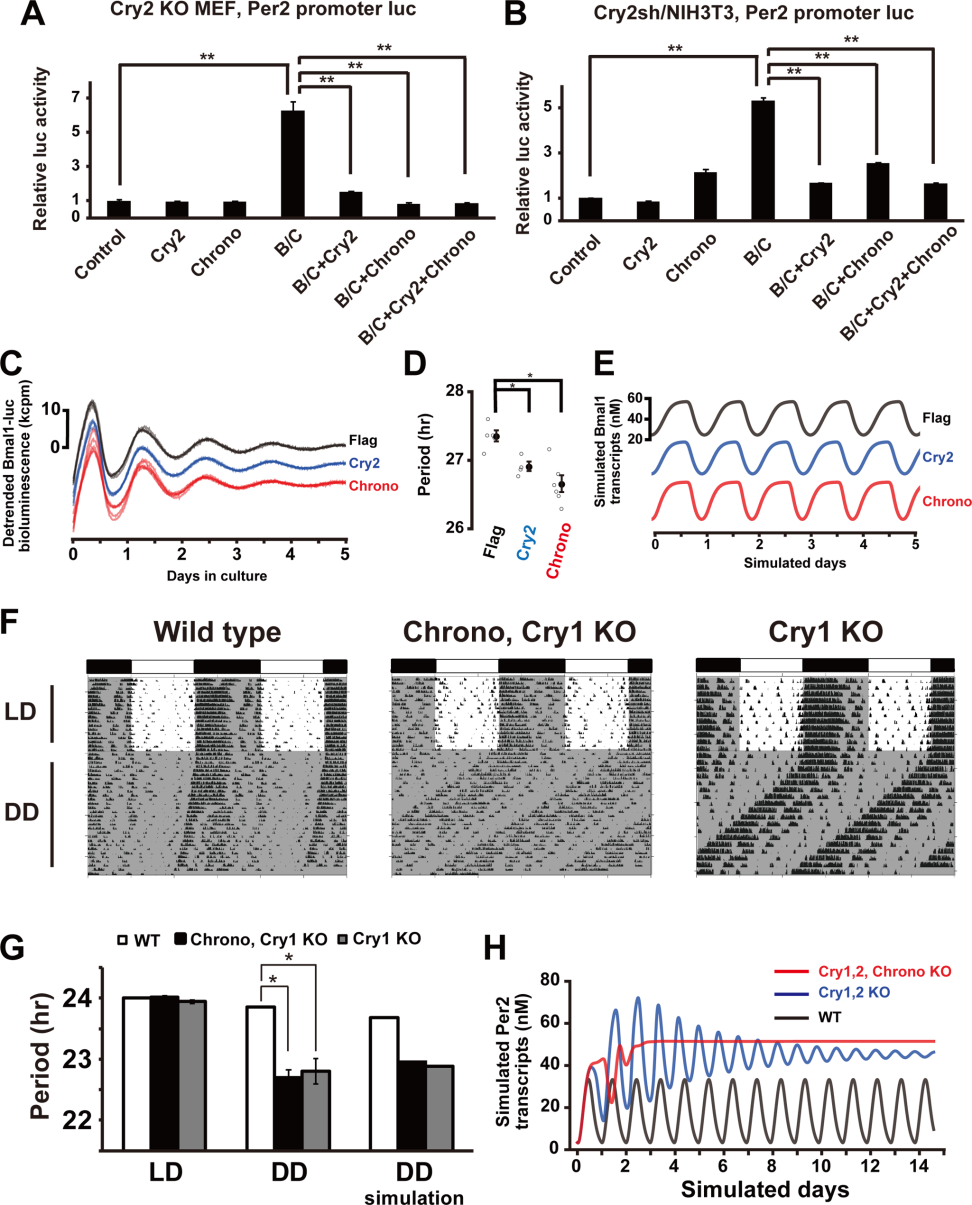
CHRONO acts as a transcriptional repressor through an independent pathway. (A and B) *Cry2* KO MEFs (A) and *Cry2*sh/NIH3T3 cells (B) were transfected with indicated combinations of expression vectors. *Chrono* inhibited BMAL1–CLOCK (B/C) complex-induced transcriptional activity in the m*Per2* promoter. The effects of overexpression of BMAL1, CLOCK, CRY2, and CHRONO proteins on *Per2* transcription were evaluated by using luciferase assays. The basal transcription activity of *Per2* promoter was set to 1. Data are means ± S.E.M. of triplicate samples. **p<0.001, Student's *t* test. (C and D) Bioluminescence rhythms (C) and periods (D) of *Cry2*sh–*Bmal1* promoter luc/NIH3T3 cells transfected with indicated expression vectors. All detrended traces (6–7 samples) from each set of experiments (Flag, *Cry2*, and *Chrono* overexpression) are superposed. The baselines are intentionally separated for each case for ease of view. The abscissa indicates the day in culture, and the ordinate the relative bioluminescence intensity (kcpm, 1,000 photon counts per minute). *Cry2*sh/NIH3T3 cells prolonged the period, and *Chrono* recovered the period length similar to *Cry2*. *p<0.005. (E) Model prediction of *Bmal1* transcript expression in the *Cry2* knockdown (Flag, black), under additional constitutive overexpression of *Cry2* (blue), and *Chrono* (red). The baselines are separated intentionally for ease of view. (F) Raw actograms of WT, *Chrono*, *Cry1* double KO and *Cry1* KO mice and comparison of free-running period estimates. A significantly shorter free-running period was observed in *Chrono*, *Cry1* double KO and *Cry1* KO mice compared to WT in DD (G, middle), in line with the prediction through *in silico* simulation (G, right). *Chrono*, *Cry1* double KO mice did not show arrhythmicity in DD. *p<0.05, Student's *t* test. (H) Model prediction of *Per2* mRNA time courses in *Cry1*, *Cry2* double KO (blue) and *Cry1*, *Cry2*, *Chrono* triple KO (red) compared to WT (black). The amounts of KO transcripts are set to 0 from the beginning of the simulated day 0, and dynamics before reaching steady state is illustrated.

*Chrono*'s role in circadian timekeeping seems to mimic that of *Cry2* both *in vivo* and in cells. Because CHRONO interacts with CRY2 and DEC2, we hypothesized that CHRONO represses BMAL1–CLOCK activity only when partnered with CRY2 or DEC2 to form a complex. To confirm this hypothesis, we performed a luciferase reporter assay using *Cry2* KO MEFs (Figure 5A) or *Cry2* knockdown (Cry2sh) NIH3T3 cells (Figures 5B and S8) and *Dec2* KO MEFs (Figure S3E). The BMAL1–CLOCK complex induced *Per2* transcription in all cells and *Chrono* repressed the BMAL1–CLOCK activity in all cells, including those without *Cry2* and *Dec2*, indicating that CHRONO acts as a transcriptional repressor via an independent pathway from CRY2 and DEC2. Moreover, we established a stable NIH3T3 cell line with *Bmal1* promoter luciferase and *Cry2* knockdown. The rhythm of fibroblasts with overexpression of Flag as a control showed a robust circadian oscillation with a lengthened period (27.29±0.06 h; mean ± S.E.M.; *n* = 4) due to *Cry2* knockdown. As expected, constitutive overexpression of *Cry2* driven by the CMV promoter partially restored the period to a shorter value (26.91±0.07 h; *n* = 4; *p<0.01, Welch's *t* test) in the *Cry2*-sh/NIH3T3 cells. Consistent with the idea that *Chrono* mimics the *Cry2* phenotype, overexpression of *Chrono* similarly shortened the period (26.66±0.15 h; *n* = 5; *p<0.01, Welch's *t* test) of oscillation in the *Cry2*-sh/NIH3T3 cells (Figure 5C and D). These relative modulations in periods are predicted in parallel by the extended Kim–Forger model (Figure 5E), under the simulated experimental conditions of *Cry2* knockdown to 30% of its original value and/or constitutive overexpression of either *Cry2* or *Chrono*. These results indicate that the circadian phenotypes of *Chrono* are similar to *Cry2*, although the repression mechanisms operate via different pathways.

Given the phenotypic similarities between *Chrono* and *Cry2*, we postulated that *Chrono* could overtake the role of *Cry2* under KO conditions. To test this idea, we generated *Chrono*, *Cry1* double KO mice by mating *Chrono* KO with *Cry1* KO mice. The *Cry1*, *Cry2* double KO mouse shows a circadian arrhythmicity in DD [28], and we expected a *Chrono*, *Cry1* double KO would exhibit similar arrhythmicity. However, the circadian locomotor activity persisted under DD. In DD condition, *Cry1* KO mice showed a shorter period than WT, similar to *Cry1*, *Chrono* double KO mice (Figure 5F). There was no significant difference of period between *Cry1* KO and *Cry1*, *Chrono* double KO mice (Figure 5G). These results are predicted by our mathematical model, which shows short period rhythms under the double *Chrono*, *Cry1* KO (22.95 h) and *Cry1* single KO (22.89 h), respectively (Figure 5G, right). The model also predicted that the *Chrono* KO does not compromise the amplitude of *Per2* mRNA rhythms (Figure 5H). Interestingly, the model predicts initially oscillating but damping *Per2* rhythms in *Cry1*, *Cry2* double KO (Figure 5H, blue). However, the triple, *Chrono*, *Cry1*, *Cry2* KO completely removed any residual oscillations (Figure 5H, red). These results led us to conclude that, although CHRONO acts as a repressor of the BMAL1–CLOCK complex just as CRY2, the mechanism of action of CHRONO is independent of CRY2 and that CHRONO may be required for rhythmicity in certain mutant backgrounds.

### *Chrono* Mediates Clock Repression in the Glucocorticoid Response

**Figure 6:**
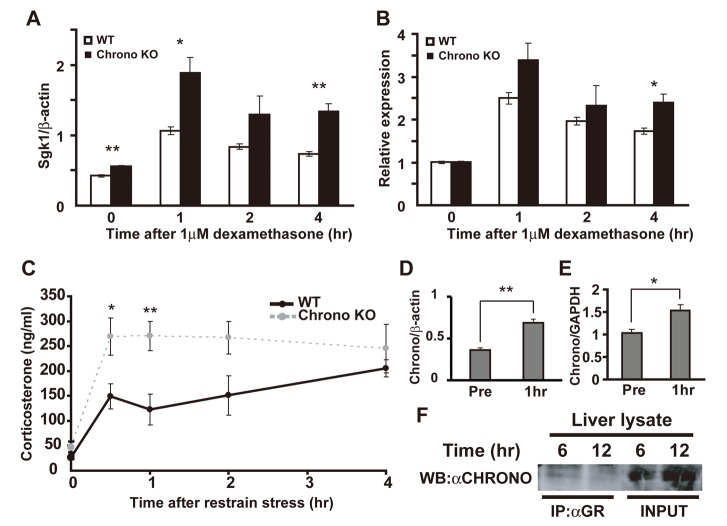
*Chrono* contributes to the repressive function in glucocorticoid response. (A) Expression of *Sgk1* in MEFs from WT and *Chrono* KO mice after treatment with dexamethasone for 1–4 h. The relative level of *Sgk1* mRNA was normalized to the corresponding *β-actin* mRNA. Data are means ± S.E.M. of triplicate samples (*p<0.05, **p<0.01, Student's *t* test). (B) The relative *Sgk1* expression level was normalized to the pretreatment (0 h) level. (C) Serum corticosterone level at 0, 0.5, 1, 2, and 4 h after restraint stress. Results are the means ± S.E.M. Asterisks indicate the existence of significant differences between the time points means within each group by one-way ANOVA followed by Tukey–Kramer's multiple comparisons test, as follows: *p<0.05 versus 0.5 h time after restraint stress; **p<0.01 versus 1 h time after restraint stress. (D) Expression of *Chrono* in WT MEFs between pre– and post–1-h treatment with dexamethasone. **p<0.01, Student's *t* test. (E) Expression of *Chrono* in hypothalamus between pre– and post–1-h restraint stress. *p<0.05, Student's *t* test. (D and E) The relative level of *Chrono* mRNA was normalized to the corresponding *β-actin* and GAPDH mRNA. (F) Endogenous CHRONO and GR interaction in the WT mouse liver at ZT 6 and 12.

The Cryptochromes (*Cry1* and *Cry2*) have been reported to mediate rhythmic repression of the GR [29]. Because *Chrono* behaves as a transcriptional repressor with an effect similar to *Cry*2, we next verified the physiological role of the *Chrono* KO against stress responses. We first examined the expression of serum/glucocorticoid-regulated kinase 1 (*Sgk1*) after dexamethasone stimulation. We found that *Sgk1* mRNA was up-regulated in *Chrono* KO MEFs when compared to WT MEFs after 1-h and 4-h exposures to dexamethasone (Figure 6A, *p<0.05, **p<0.01, Student's *t* test). The relative expression of *Sgk1* was also activated by 4-h exposure to dexamethasone (Figure 6B; *p<0.05, Student's *t* test). Serum corticosterone levels *in vivo* were also robustly increased in *Chrono* KO when compared with WT mice after 0.5 h and 1 h of restraint stress (Figure 6C; *p<0.05; one-way ANOVA followed by Tukey–Kramer's multiple comparisons test, as follows, *p<0.05 versus 0.5 h time after restraint stress; **p<0.01 versus 1 h time after restraint stress). In a WT background, *Chrono* mRNA was up-regulated in MEFs (Figure 6D; **p<0.01, Student's *t* test) and hypothalamus (Figure 6E; *p<0.05, Student's *t* test) after 1 h of exposure to dexamethasone and restraint stress, respectively, whereas *Cry2* mRNA was not significantly increased under restraint stress in both WT and *Chrono* KO hypothalamus as well as MEFs (Figure S9). Furthermore, the IP-Western experiment indicated CHRONO endogenously interacted with GR *in vivo* (Figure 6F). These results suggest that *Chrono* is up-regulated by stress-dependent behavior and contributes to repressive function in the glucocorticoid response.

### *Chrono* in the SCN Plays a Central Role in Behavioral Rhythms

**Figure 7:**
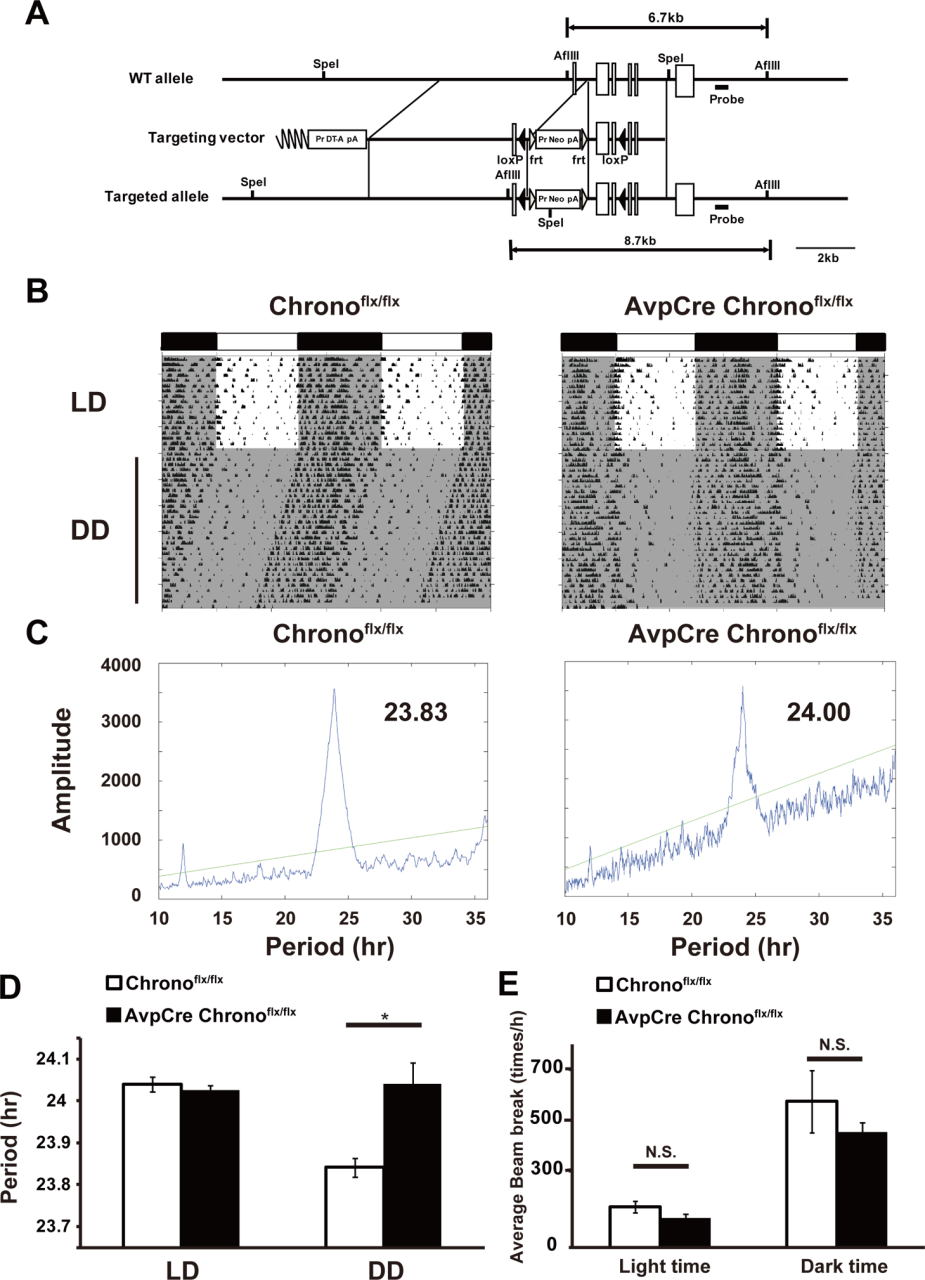
*Chrono* in the *Avp* neurons plays a central role in behavioral rhythms. (A) The targeting strategy is illustrated with the structure of the WT *Chrono* allele, the targeting vector, the floxed allele *Chrono* ^flx^. *Chrono* exons are shown as open boxes; 5′ genomic DNA, intronic sequences, and 3′ genomic DNA as solid lines. The Pr Neo pA and the Pr DT-A pA cassettes are indicated as open boxes. The loxP sequences are shown as triangles. (B) Representative actograms of *Chrono* ^flx/flx^ and *Avp*Cre *Chrono* ^flx/flx^ mice. Animals were maintained on 12–12 h LD cycles initially and transferred to DD. Shaded area indicates dark phase. (C) Representative chi-squared periodograms of *Chrono* ^flx/flx^ and *Avp*Cre *Chrono* ^flx/flx^ locomotor activities for 1 wk under DD. (D) Comparison of free-running periods estimated for *Chrono* ^flx/flx^ and *Avp*Cre *Chrono* ^flx/flx^ mice. Pairwise comparisons indicated that there were no significant differences in circadian periods under LD compared with the *Chrono* ^flx/flx^ littermates. However, a significantly longer free-running period was observed in *Avp*Cre *Chrono* ^flx/flx^ mice compared with the *Chrono* ^flx/flx^ littermates in DD. *Avp*Cre *Chrono* ^flx/flx^ mice (24.04±0.12 h, *n* = 6, male) and *Chrono* ^flx/flx^ siblings (23.84±0.06 h, *n* = 8, male) in DD (mean period ± S.D.; *p<0.01, Student's *t* test). (E) Basal locomotor activity. Amounts of activities during the light phase (left) and the dark phase (right) for 1 wk in LD show no significant differences. *n* = 8 for *Chrono* ^flx/flx^ and *n* = 6 for *Avp*Cre *Chrono* ^flx/flx^.

To identify the role of *Chrono* in the SCN, we generated mice carrying a conditional *Chrono* KO allele using the Cre-loxP system (Figures 7A and S10). *Avp*-Cre mice express Cre specifically in arginine vasopressin (AVP) neurons (GENSAT project) [30]. AVP is the most abundant neuropeptide in the hypothalamus and specifically in the SCN [31],[32]; therefore, *Avp*-Cre *Chrono* ^flx/flx^ mice can be considered as SCN-targeted *Chrono* KO mice. The average locomotor activity rhythm of *Avp*-Cre *Chrono* ^flx/flx^ male mice (24.04±0.12 h, *n* = 6) was significantly longer than that of *Chrono* ^flx/flx^ littermates (23.84±0.06 h, *n* = 8) (*p<0.01, Student's *t* test) (Figure 7B–D). The basal locomotor activity was measured during the light phase and the dark phase after 1 wk in LD by the infrared beam breaking. The activity amount of the *Avp*-Cre *Chrono* ^flx/flx^ mouse was not altered compared with *Chrono* ^flx/flx^ mouse (Figure 7E). These results demonstrate that *Chrono* expression in the *Avp* neurons plays a central role in behavioral rhythms.

## Discussion

In this study, we characterized a gene called *Chrono* that serves as a component in the negative arm of the core mammalian circadian clock. Indeed, based on our genomic analysis of circadian promoters, CHRONO appears to be one of the last remaining components of the clock. The most interesting feature of CHRONO is its regulation by epigenetic control and behavioral stress. These findings place CHRONO in a central position to couple stress metabolism to clock regulation.

Our results suggest that CHRONO operates as a repressor of the core circadian feedback loop through the recruitment of HDAC. The repressive effects of CHRONO were also seen in *Cry2* KO MEFs and *Dec2* KO MEFs, which shows that CHRONO repression does not require cooperative interactions with CRY2 or DEC2 [33],[34]. It has been reported that the recruitment of histone-modifying enzymes is regulated in a circadian manner [35]–[39] and some complexes are observed in transcriptional repression as in PER and SIN3–HDAC [14]. HDAC1 rhythmically bound to the *Per2* promoter (Figure S3F), and this result was consistent with a recent report [40]. Acetyl-Histone H3 occupancy around E-boxes was enhanced in *Chrono* KO MEFs compared to WT (Figure S7E and F), suggesting that *Chrono* also has the potential to form complexes with other histone-modifying enzymes. However, HAT (histone acetyltransferase) activity by CLOCK was not affected by CHRONO *in vitro* experiments (Figure S11). Further mechanistic study is required. CHRONO endogenously binds to the E-boxes of circadian genes with circadian rhythmicity, suggesting that *Chrono* is a core circadian repressor that operates as an auto-regulated component of the clock.

The *Chrono* KO mouse showed a lengthened circadian period similar to the *Cry2* KO mouse [28]. Overexpression of *Chrono* restored the period of *Bmal1-luc* oscillations in *Cry2* knockdown cells in a manner comparable to *Cry2* overexpression. On the other hand, the *Chrono*, *Cry1* double KO mouse did not show arrhythmic behavior as seen in the *Cry1*, *Cry2* double KO mouse [28]. Together, these results suggest that *Chrono* plays a similar but independent role from *Cry2*. A conditional *Chrono* KO mouse driven by the *Avp* promoter (*Avp*Cre *Chrono* ^flx/flx^) also showed a lengthened circadian period. This points to *Chrono*'s central role in the core clock and that its expression in *Avp* neurons is critical for the determination of behavioral circadian period. It also demonstrates that the *Avp*Cre system (GENSAT line number QZ20_CRE) can potentially prove useful in dissecting the output pathway of the SCN that may perform coding by internally distributed periods [41].

The role of *Chrono* was also tested *in silico* by modeling mechanisms of *Chrono*-mediated repression of BMAL1–CLOCK (Figure 2D) within the most comprehensive and realistic mathematical model of the mammalian circadian clock [20]. The extended model successfully predicted that the reduced (or enhanced) *Chrono* expression results in a lengthened (or shortened) period (Figures 4D and 5E), matching the phenotypes of *Chrono* KO or overexpression (Figures 4D and 5D). This indicates that the proposed biochemical mechanisms of *Chrono*-mediated repression of BMAL1–CLOCK (Figure 2D) are consistent with the overall phenotypes observed when *Chrono* levels are changed.

*Chrono* is likely to be a physiological response-dependent regulator. In response to dexamethasone application, *Sgk1* was up-regulated in *Chrono* KO MEFs when compared with WT MEFs. *In vivo* serum corticosterone levels were also increased in the *Chrono* KO mouse when compared with the WT mouse under restraint stress and CHRONO itself interacted with the GR. Along with the observation that *Chrono* mRNA expression was induced by the stress response in MEFs and hypothalamus, we conclude that *Chrono* is a potential repressor activated by behavioral stress and can couple the clock with the hypothalamic–pituitary–adrenal (HPA) axis. Our previous results showed acute physical stress also elevated *Per1* mRNA through a glucocorticoid-responsive element [42]. Further experimental work is needed to reveal the detailed molecular interactions between the circadian clock and the HPA axis.

Our discovery of the role of *Chrono* opens up many possibilities for future work. Our mathematical modeling raises the interesting possibility that the *Per2* oscillations from *Cry1*, *Cry2* double KO neonatal SCN [25] is mediated by *Chrono*. In further studies, it would be interesting to see if the *Cry1*, *Cry2*, *Chrono* triple KO eliminates the ability to oscillate in neonatal SCNs as predicted by our model (Figure 5H). Further work should also examine the role of *Chrono* in linking the HPA to the circadian clock. Taken together, we conclude that *Chrono* is a novel circadian clock gene that acts as a repressor in the circadian system and modulates physiology.

## Materials and Methods

### Ethics Statement

All protocols of animal experiments followed in the present study were approved by the Animal Research Committee of Hiroshima University and Animal Care and Use Committees of the RIKEN Brain Science Institute.

### Cell Culture

NIH3T3, COS7 cells and MEFs were maintained in Dulbecco's Modified Eagle Medium (DMEM; Nacalai Tesque, Kyoto, Japan) supplemented with 10% fetal bovine serum (FBS) and penicillin–streptomycin antibiotics at 37°C and 5% CO_2_.

### Chromatin IP

NIH3T3 and MEF cells were stimulated with 100 nM dexamethasone containing medium at each time point. Cells were fixed in 1× PBS containing 0.5% formaldehyde. Glycine was added to a final concentration of 0.125 M, and the incubation was continued for an additional 15 min. After washing the samples with ice-cold phosphate-buffered saline, the samples were homogenized in 1 mL of ice-cold homogenize buffer (5 mM PIPES [pH 8.0], 85 mM KCl, 0.5% NP-40, and protease inhibitors cocktail) and centrifuged (15,000× *g*, 4°C, 5 min). The pellets were suspended in nucleus lysis buffer (50 mM Tris-HCl [pH 8.0], 10 mM EDTA, 1% SDS, protease inhibitors) and sonicated 20 times for 30 s each time at intervals of 60 s with a Bioruptor (Diagenode, Inc.) or sonicated 10 times for 10 s each time at intervals of 50 s with a MICROSON (Misonix, Inc.) for brain samples. The samples were centrifuged at 15,000 rpm at 4°C for 5 min. Supernatants were diluted 10-fold in ChIP dilution buffer (50 mM Tris-HCl [pH 8.0], 167 mM NaCl, 1.1% Triton X-100, 0.11% sodium deoxycholate, protease inhibitor).

### Chromatin IP Sequencing (ChIP-seq)

Whole brain samples from mice (C57BL/6J) were used for ChIP-seq. BMAL1-bound DNA was purified by SDS-PAGE to obtain 150–200 bp fragments and sequenced on an Illumina GA sequencer at the Research Center of Research Institute for Radiation Biology and Medicine (RiRBM), Hiroshima University. We generated 15,000–20,000 clusters per “tile,” and 26 cycles of the sequencing reactions were performed according to the manufacturer's instructions. The identification of each DNA fragments was performed using Genome Studio software (Illumina Inc.).

### ChIP-PCR

CHRONO, BMAL1, HDAC1 (ab7028, Abcam), Acetyl-Histone H3 (06-599, Millipore), and IgG-bound DNA were used for quantitative real-time reverse-transcription PCR (RT-PCR). The primers were designed for amplifying the E-box-like regions in *Per2* and *Dbp* promoters.

### *In Vitro* Real-Time Oscillation Monitoring System (IV-ROMS)

NIH3T3 cultures at the concentration of 1×10^5^ cells in Opti-MEM (Gibco) supplemented with 10% FBS in a 35 mm dish were transfected with the desired plasmids by using the Lipofectamine reagent (Invitrogen). The medium was exchanged 24 h after transfection with 100 nM dexamethasone containing medium, and 2 h later this medium was replaced with Opti-MEM supplemented with 1% FBS and 0.1 mM luciferin–10 mM HEPES (pH 7.2). Bioluminescence was measured by using the IV-ROMS (Hamamatsu Photonics) as described previously [26],[43].

### Luciferase Assay

NIH3T3 cells and MEFs were cultured and transfected with the desired plasmids by using Lipofectamine 2000 (Invitrogen) or Nucleofection (Lonza). Cells were harvested 24 h after transfection, and cell lysates were prepared and then used in the dual luciferase assay system (Promega). For exogenous expression, we transfected cells with pcDNA3 driven by ubiquitous cytomegalovirus (CMV) promoter.

### Western Blotting and IP

Rabbit antibody against HA-tag and mouse antibodies against Flag-tag and Myc-tag were subjected to Western blot according to the manufacturer's protocol. For IP, COS7 cells transfected with the desired plasmids by using Lipofectamine 2000 (Invitrogen) were lysed in TNE buffer with protease inhibitor. Following the standard protocol, lysates were precleared with Dynabeads Protein G (Invitrogen) and then immunoprecipitated with rabbit anti-HA antibodies (Cell signaling), mouse anti-Flag antibodies (Sigma), or mouse anti–c-Myc antibodies (Sigma). After washing three times, the precipitates were resuspended in the 2× SDS-PAGE sample buffer, boiled for 5 min, and run on a 10% SDS-PAGE gel followed by Western blot analysis. Immunoreactive bands were detected by ODYSSEY Infrared Imaging System (LI-COR). These experiments were independently performed three times. The intensity of the band (BMAL1 and CHRONO rpar; was calculated by using ODYSSEY Infrared Imaging System.

### Animal Care and Behavioral Analysis

Circadian rhythms of locomotor activity were analyzed as previous described [43]. Each mouse was individually housed for 2 wk in 12–12 h LD cycles, and then for 4 wk in constant darkness (DD). Locomotor activity was monitored with a locomotor activity recording apparatus (Biotex, Kyoto, Japan) that measures events of infrared beam breaking in 1-min bins. The data from the first week under DD were used to estimate the period of circadian locomotor activities of WT and *Chrono* KO mice. For RNA sampling, dissected tissues were immediately frozen in liquid nitrogen and stored at −80°C until processing.

### Methods for Light Treatment

The experiment was performed as described previously [44]. Male ICR mice (SLC, Japan) were exposed to an incandescent light stimulus (1,000 lux, 30 min) at CT16 in the second DD cycle. Animals were sacrificed 60 min after the initiation of the light exposure. Total RNA was prepared from six pooled pairs of SCN at each time point using PicoPure RNA Isolation kit (Applied Biosystems).

### *In Vitro* Translation

CHRONO protein was synthesized using the TNT T7 Coupled Reticulocyte Lysate System (Promega). We added 2 µg of template DNA (Chrono/pcDNA3 or Chrono–Flag/pcDNA3) to an aliquot of the TNT Quick Master Mix and incubated it in a 50 µl reaction volume for 60–90 min at 30°C. Synthesized proteins were detected by 10 mCi/ml (specific activity, 30 TBq/mmol) [^35^S]methionine, and resolved on SDS-PAGE (10%) gels using one-fifth of each translation reaction product mixed with an equal volume of sample buffer (15% glycerol, 5% β-mercaptoethanol, 4.5% SDS, 100 mM Tris-Cl, pH 6.8, 0.03% bromophenol blue). Gels were fixed, dried, and exposed to Hyper-film (Amersham) for 16 h to 2 d. The molecular masses (in kDa) of the translated proteins were derived using standard curves generated from protein size standards (Bio-Rad).

### Quantitative RT-PCR

Each quantitative real-time RT-PCR was performed using the ABI Prism 7900HT sequence detection system as described previously [42],[45]. The PCR primers were designed with the Primer Express software (Applied Biosystems). The reaction was first incubated at 50°C for 2 min and then at 95°C for 10 min, followed by 40 cycles at 95°C for 15 s and 60°C for 1 min.

### Corticosterone Level Under the Restraint Stress

Blood samples were collected by tail bleeding at time points of 0, 0.5, 1, 2, and 4 h after restraint stress. Corticosterone in mouse serum was measured using YK240 corticosterone enzyme immunoassay (EIA) kit (Yanaihara Institute).

### RNAi Experiment

BLOCK-iT Lentiviral RNAi System (Invitrogen) was used for RNAi experiments. NIH3T3 cells at the density of 5×10^4^ were infected with a lentiviral vector and cultured. We selected stably transfected cells with zeocin. The shRNA/NIH3T3 cells were infected with *Bmal1* promoter-driven luciferase lentiviral vector and selected for stable expression with blasticidin.

### Antibodies

Antibody against BMAL1 was generated as described previously [22]. Purified glutathione S-transferase (GST) –*Gm129* N-terminal (amino acids 1 to 187) protein was produced as a recombinant protein in the competent cells BL21 (DE3) (Stratagene). After removing GST by using GSTrap FF and PreScision Protease (GE Healthcare), the produced antigen was used to immunize rabbits. The antiserum was subjected to affinity purification using Affi gel 10 (Bio-Rad) conjugated with the antigen. The anti-CHRONO antibody recognized its target protein in immunochemical analysis (Figure S4D).

### *Chrono* (*Gm129*) Gene Trap Mice

C57BL/6 gene trap ES cell clone IST11761C7 was an embryonic stem cell provided by the gene trap method [46]. Long terminal repeat (LTR) –splice acceptor–βgeo–polyA–LTR sequence was inserted between exons 1 and 2 in one allele of the *Chrono* (*Gm129*) gene (Figure 4A) (TIGM, Texas A&M Institute for Genomic Medicine). In substitution for mRNA producing CHRONO protein, mRNA producing fusion protein of the neomycin-resistant gene product and β-galactosidase (B-geo) was transcribed from this variation allele. We generated chimeric mice from C57BL/6 gene trap ES cell clone IST11761C7, mated the chimeric mice with WT C57BL/6 (+/+), and subsequently obtained heterozygous KO mice (+/−). Ultimately, we obtained homozygous KO (−/−) mice by mating heterozygous KO mice. Absence of *Chrono* mRNA and CHRONO protein was confirmed by PCR, RT-PCR, and Western blotting (Figure S4B–D).

### mRNA Expression of Core Circadian Genes in *Chrono* KO MEFs and Liver

WT and *Chrono* KO MEFs were stimulated with 100 nM dexamethasone containing medium. After 32 h, mRNA were extracted by TRI reagent (Molecular Research Center, Inc.) every 4 h. Quantitative real-time RT-PCR was described above. Adult WT and *Chrono* KO mice were exposed to 2 wk of LD cycles and then kept in complete darkness as a continuation of the dark phase of the last LD cycle. mRNA expression was examined CT12 and next CT0 from the third DD cycle.

### Generation of *Chrono* Conditional KO Mice

The *Chrono* conditional KO mice (Accession No. CDB0913K; http://www.cdb.riken.jp/arg/mutant%20mice%20list.html) were generated as described (http://www.cdb.riken.jp/arg/Methods.html). To generate a targeting vector, genomic fragments of the *Chrono* locus were obtained from the RP23-385O12 BAC clone (BACPAC Resources). A 950 bp region containing exons 2 and 3 of the *Chrono* gene was flanked by loxP sites (Figure 7A). Targeted ES clones were microinjected into ICR eight-cell stage embryos, and injected embryos were transferred into pseudopregnant ICR females. The resulting chimeras were bred with C57BL/6 mice, and heterozygous offspring were identified by PCR. Primers for 5′ loxP were used—forward E1F (5′-CAGACAGTGAAGAAGCTGCATA-3′) and reverse loxR2 (5′-CAGACTGCCTTGGGAAAAGC-3′)—yielding no product for WT allele and 603 bp products for the targeted allele, respectively. Primers for 3′ loxP were used—forward loxF1 (5′-GGCATGGGCTATTCTGTTTG-3′) and reverse loxR1 (5′-TTGAGGGAAACAGCAGAGGT-3′)—yielding 121 bp products for WT allele and 179 bp products for the targeted allele, respectively (Figure S10A and C).

### *In Silico* Evaluation of *Chrono* in the Oscillator Network

We incorporated *Chrono* into a recently developed mathematical model of the mammalian circadian clock [20] based on our biochemical findings. We assumed that the mRNA degradation rate of *Chrono* is the same as *Per2*, as the *Chrono* mRNA time course is similar to *Per2* mRNA [42]. We further assumed the time course of PER2–CHRONO nuclear entry is similar to the PER–CRY complexes. However, as found in our experiments, the CHRONO protein binds to the PER2 protein but not to PER1 protein (Figure 2A) and that the CHRONO protein interacts with BMAL1–CLOCK to inhibit its E-box activation on *Per1*, *Per2*, *Cry1*, *Cry2*, and *Rev–erb*s promoters. In the model, we did not consider binding between CHRONO and CRY2, as BMAL1–CLOCK repression by CHRONO did not depend on the presence of CRY2 (Figure 5). In total, 38 variables were newly added to the Kim–Forger model, which account for all the possible complexes involving CHRONO (details of computer simulation are described in Text S1, Tables S1, S2, S3, and Appendix S1). All the simulations were performed with Mathematica 8.0 (Wolfram Research).

## Supporting Information

Figure S1 **Sequence conservation of** ***Chrono*** **.** The protein sequence alignment of *Chrono* across species was performed with Homologene database (NCBI). *Chrono* is highly conserved in mammals.
(TIF)

Figure S2 **Characterization of CHRONO.** (A–C) CHRONO protein expression showed circadian rhythm antiphasic to BMAL1 in the liver. We prepared liver samples at CT 2, 8, 14, and 20. Each time point has four or five samples, which were dissolved with RIPA buffer. The *x*-axis represents time in CT and *y*-axis protein amounts. The relative levels of protein were normalized to the β-ACTIN protein levels. The maximum protein amount was set to 100. (D) A schematic model of CHRONO interaction. CHRONO interacts with BMAL1, PER2, CRY2, and DEC2 (red line), but not PER1, CRY1, and DEC1 (see Figure 2A and B).
(TIF)

Figure S3 ***Chrono*** **represses transcriptional activity.** (A) Effects of *Chrono* expression on the *Per2* promoter luciferase activities. *Chrono* repressed the *Per2* transactivation by BMAL1 and CLOCK (B/C) overexpression with the potency similar to *Dec2*. The bar plots represent means ± S.E.M. of four samples (*p<0.05, Student's *t* test). (B) Effects of *Chrono* expression on the *Chrono* promoter luciferase activity. Overexpressed CHRONO repressed transactivation by exogenous expression of BMAL1 and CLOCK (B/C) in *Chrono* promoter, same as *Cry2*. Bars represent means ± S.E.M. of four samples (*p<0.05, **p<0.001, Student's *t* test). (C) Effects of *Chrono* and *Cry2* expression on the *Dbp* promoter luciferase activities. *Chrono* repressed the *Dbp* promoter activity with the potency similar to *Cry2*. The abscissa indicates the day in culture, and the ordinate the relative bioluminescence intensity (kcpm, 1,000 photon counts per minute). The first amplitude (D) was significantly decreased with overexpression of *Chrono* compared to control. The bar plots indicate the mean ± S.E.M (shaded area) of eight samples. **p<0.0001, Student's *t* test. (E) *Dec2* KO MEFs were transfected with combinations of expression vectors as indicated. *Chrono* inhibited BMAL1–CLOCK complex-induced transcriptional activity on the m*Per2* promoter. The effects of overexpression of BMAL1, CLOCK, DEC2, and CHRONO proteins on *Per2* transcription were evaluated by measuring bioluminescence from luciferase activities. The basal transcription level of the *Per2* promoter was set to 1. The bar plots indicate the mean ± S.E.M of triplicate samples. *p<0.001, Student's *t* test. (F) ChIP analyses for HDAC1 and IgG (negative control). The HDAC1 occupancies at the endogenous E-box of the *Per2* promoter were detected in the WT MEF cells at 28, 36, 44, and 52 h after induction with dexamethasone. The graph showed relative real-time PCR values. The maximum value of WT was set to 100. Data are means ± S.E.M. of three samples. (F) ChIP analysis for BMAL1 and IgG (negative control). The BMAL1 occupancy at the endogenous E-box of *Per2* promoter was detected in NIH3T3 cells after 100 nM dexamethasone stimulation. The graph showed relative real-time PCR values. The data were plotted as percentages relative to the input DNA. Data are means ± S.E.M. of 3–4 samples.
(TIF)

Figure S4 **Construction of** ***Chrono*** **KO mice.** (A) The targeting strategy for PCR genotyping. An LTR–splice acceptor–βgeo–polyA–LTR sequence is inserted between exons 1 and 2 of *Chrono* allele (TIGM, Texas A&M Institute for Genomic Medicine). Primer locations are schematically displayed in (A). (B) PCR genotyping of DNA extracted from mouse tails of KO (*Chrono* ^−/−^), heterozygous (*Chrono* ^+/−^), WT (*Chrono* ^+/+^) offspring. (C) RT-PCR analysis of *Chrono* expression in the hypothalamus. (D) Western blot analysis of *Chrono* expression in the liver of KO (*Chrono* ^−/−^) and WT (*Chrono* ^+/+^).
(TIF)

Figure S5 **The simulated time courses of** ***Chrono*** **mRNA and** ***Cry2*** **mRNA in the mathematical model.** The amplitude and phase of *Chrono* mRNA and *Cry2* mRNA are very different, matching experimental data [22],[47]. That is, the amplitude of the *Chrono* mRNA rhythm is much larger than that of *Cry2* mRNA, and the phase of the *Chrono* mRNA rhythm is more advanced than that of *Cry2* mRNA.
(TIF)

Figure S6 **Characterization of** ***Chrono*** **KO and light response of** ***Chrono in vivo*** **.** (A) Representative actograms from WT and *Chrono* KO mice that were subjected first to LD cycles, followed by a 6-h jet-lag light phase advance. Shaded areas indicate the dark phase. (B) Re-entrainment traces from an average of WT (red), *Chrono* KO (green), and merged (right). (C) Although a 30 min light pulse (1,000 lux) delivered from CT16.0 to CT16.5 induced *Per1* mRNA expression (*p<0.05, Student's *t* test), *Chrono* expression was not induced.
(TIF)

Figure S7 **Characterization of** ***Chrono*** **KO and Chrono may change the epigenetic modification of cells via Acetyl-Histone H3.** Analysis of mRNA of core clock genes in WT and *Chrono* KO MEFs. Temporal mRNA expression of *Chrono* (A) and *Per2* (B) in WT and *Chrono* KO MEFs. The abscissa represents time after dexamethasone stimulation and the ordinate the mRNA amounts. (C) mRNA expressions of circadian genes in WT and *Chrono* KO liver at CT12 and CT0. The relative levels of mRNA were normalized to the corresponding GAPDH mRNA levels. Plots and error bars represent mean ± S.E.M. of four samples. *p<0.05, **p<0.01, Student's *t* test. (D) Expression patterns of circadian genes in the SCN of WT and *Chrono* KO mice. The SCN samples from four or five mice were mixed at each time point. Solid lines with white circles and dotted lines with black circles represent WT and *Chrono* KO, respectively. The relative levels of each mRNA are normalized to the corresponding GAPDH RNA level. (E and F) ChIP analysis for Acetyl-Histone H3. The Acetyl-Histone H3 occupancies at the endogenous E-boxes of *Per2* (E) and *Dbp* (F) promoters were detected in WT and *Chrono* KO MEFs at 52 h after induction with dexamethasone. The data were plotted as percentages relative to the input DNA. Data are means ± S.E.M. of five samples.
(TIF)

Figure S8 **RT-PCR quantification of** ***Cry2*** **mRNA abundance in NIH3T3 fibroblasts that stably express the** ***Cry2*** **shRNA.** LacZsh was used as a control. The relative levels of mRNA were normalized to the corresponding GAPDH mRNA levels. The maximum mRNA amount was set to 100. *p<0.01, Student's *t* test.
(TIF)

Figure S9 **Expression of** ***Cry2*** **in hypothalamus under restraint stress.** Expression of *Cry2* in hypothalamus under restraint stress (pre- and after 1 h) of WT (A) and *Chrono* KO mice (B). The relative level of *Cry2* mRNA was normalized to the corresponding GAPDH mRNA. Expression of *Cry2* in WT MEFs after Dex stimulation (C) (pre- and after 1 h).
(TIF)

Figure S10 **Construction of** ***Avp*** **-specific KO of** ***Chrono*** **in mice.** (A) The targeting strategy for PCR genotyping illustrated by the structures of the WT *Chrono* allele and the floxed allele *Chrono* ^flx^. *Chrono* exons are represented as open boxes and 5′ genomic DNA, intronic sequences, and 3′ genomic DNA as solid lines. The Pr Neo pA and the Pr DT-A pA cassettes are shown as open boxes. The loxP sequences are indicated as solid triangles and the frt sequences as open triangles. (B) Southern blot verification of mouse tail DNA containing the *Chrono* ^flx^ allele. Extracted DNA samples were digested with AflIII and hybridized with the probe (Figure 7A). The WT (6.7 kb) and *Chrono* ^flx^ (8.7 kb) alleles were detected. (C) Mouse tail DNA from mice carrying WT and/or the *Chrono*flx allele were genotyped by PCR using primer sets E1F/loxR2 or loxF1/loxR1, as shown in (A). E1F/loxR2 amplifies a fragment of 603 bp only from the *Chrono* ^flx^ allele. loxF1/loxR1 amplifies fragments of 121 bp from the WT allele and 179 bp from the *Chrono* ^flx^ allele.
(TIF)

Figure S11 **HAT activity in NIH3T3.** HAT activity in NIH3T3 cells, which were transfected with the desired plasmids by using Lipofectamine 2000 (Invitrogen). After 24 h from transfection, the nuclear protein was extracted and the Histone acetyltransferase activity was measured by using HAT assay kits (ab65352, Abcam).
(TIF)

Table S1 **Newly added variables for mRNA dynamics of** ***Chrono*** **.**
(DOCX)

Table S2 **Extended variables of protein complexes.**
(DOCX)

Table S3 **Newly added/modified parameters for** ***Chrono*** **dynamics.**
(DOCX)

Text S1 **Supplementary methods.**
(DOCX)

Appendix S1 **Newly added and modified equations.**
(DOCX)

## Abbreviations

AVP: arginine vasopressin
ChIP: chromatin immunoprecipitation
CT: circadian time
DD: dark–dark
GR: glucocorticoid receptor
HAT: histone acetyltransferase
HDAC: histone deacetylase
IP: immunoprecipitation
KO: knockout
LD: light–dark
MEF: mouse embryonic fibroblast
SCN: suprachiasmatic nucleus
TSA: trichostatin A
TTFL: transcription–translation feedback loop
WT: wild–type
