# Supplementary figures and images for: A Novel Protein, CHRONO, Functions as a Core Component of the Mammalian Circadian Clock
Source: PLoS Biol. 2014 Apr 15;12(4):e1001839. doi: 10.1371/journal.pbio.1001839 (PMC3988004; doi:10.1371/journal.pbio.1001839)

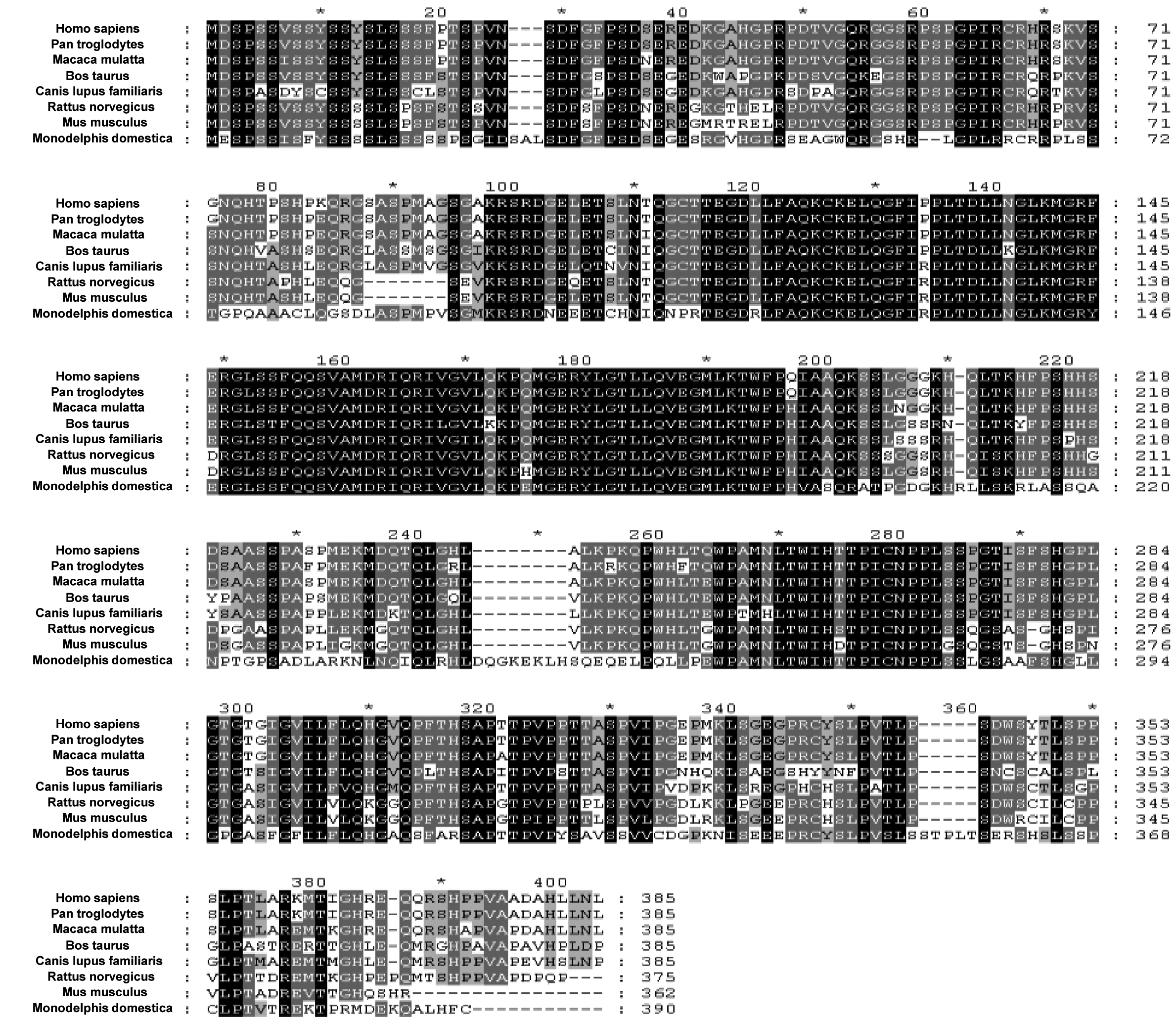

Supplement: Figure S1 — Sequence conservation of Chrono . The protein sequence alignment of Chrono across species was performed with Homologene database (NCBI). Chrono is highly conserved in mammals. (TIF) [file pbio.1001839.s001.tif]

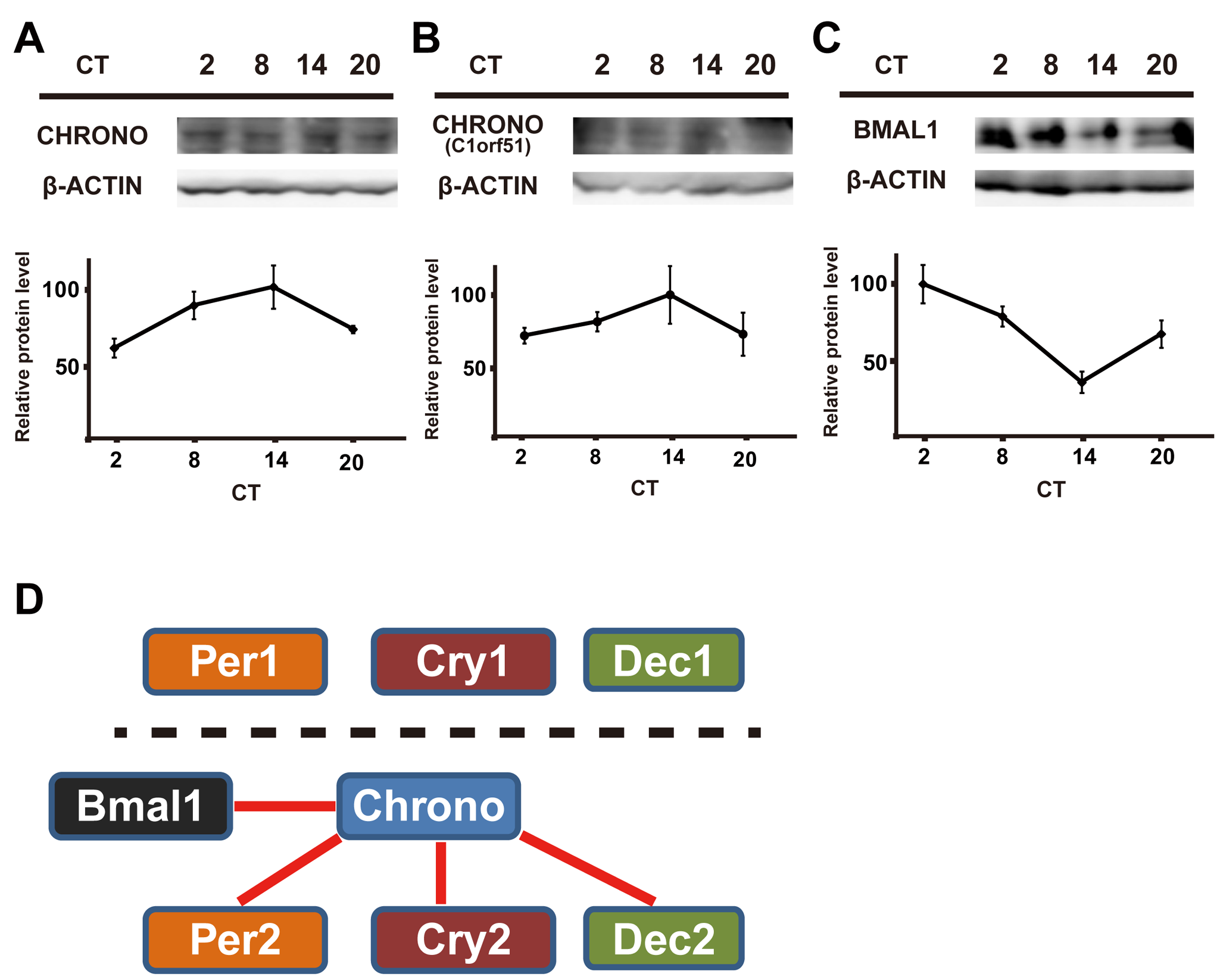

Supplement: Figure S2 — Characterization of CHRONO. (A–C) CHRONO protein expression showed circadian rhythm antiphasic to BMAL1 in the liver. We prepared liver samples at CT 2, 8, 14, and 20. Each time point has four or five samples, which were dissolved with RIPA buffer. The x-axis represents time in CT and y-axis protein amounts. The relative levels of protein were normalized to the β-ACTIN protein levels. The maximum protein amount was set to 100. (D) A schematic model of CHRONO interaction. CHRONO interacts with BMAL1, PER2, CRY2, and DEC2 (red line), but not PER1, CRY1, and DEC1 (see Figure 2A and B). (TIF) [file pbio.1001839.s002.tif]

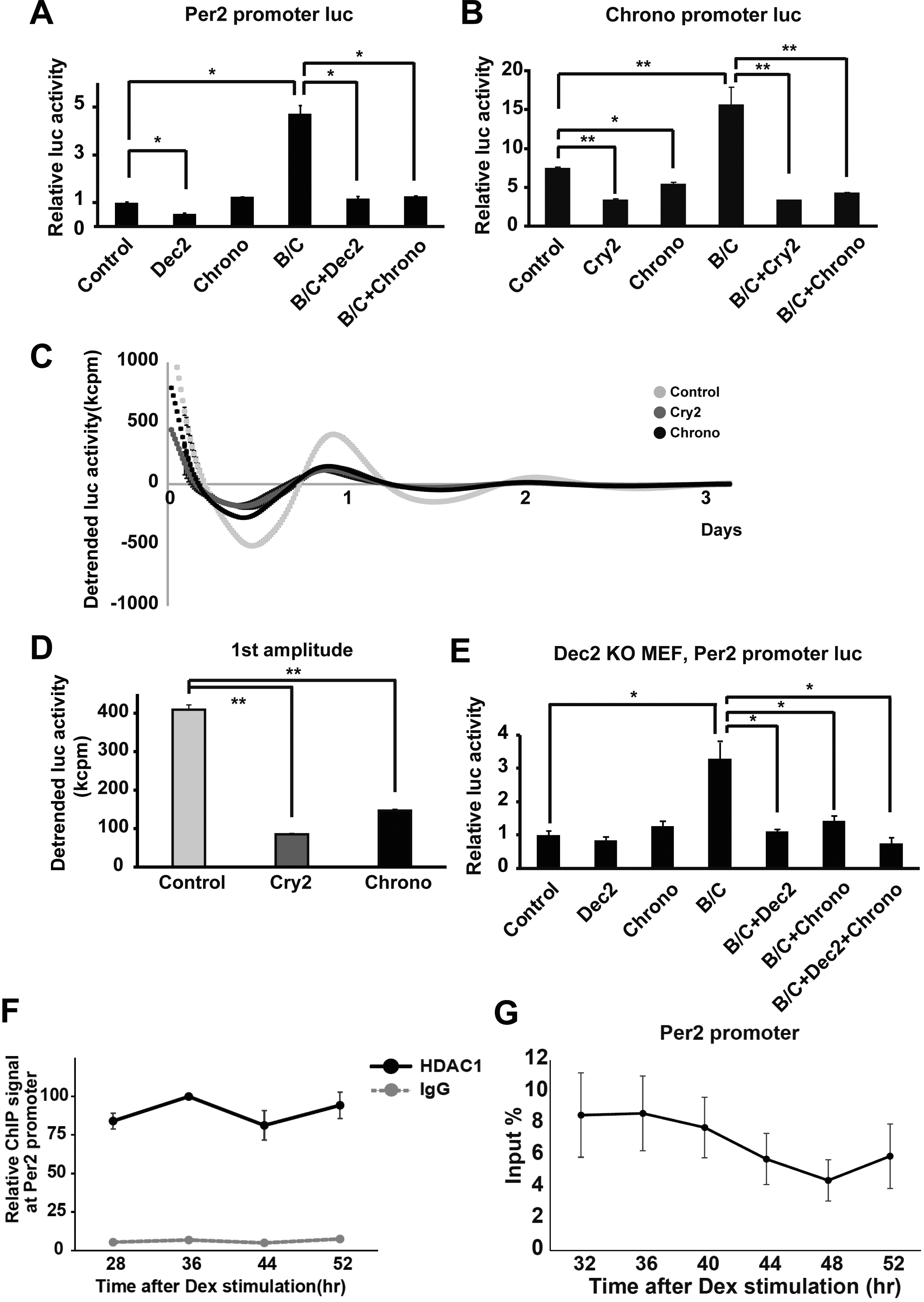

Supplement: Figure S3 — Chrono represses transcriptional activity. (A) Effects of Chrono expression on the Per2 promoter luciferase activities. Chrono repressed the Per2 transactivation by BMAL1 and CLOCK (B/C) overexpression with the potency similar to Dec2. The bar plots represent means ± S.E.M. of four samples (*p<0.05, Student's t test). (B) Effects of Chrono expression on the Chrono promoter luciferase activity. Overexpressed CHRONO repressed transactivation by exogenous expression of BMAL1 and CLOCK (B/C) in Chrono promoter, same as Cry2. Bars represent means ± S.E.M. of four samples (*p<0.05, **p<0.001, Student's t test). (C) Effects of Chrono and Cry2 expression on the Dbp promoter luciferase activities. Chrono repressed the Dbp promoter activity with the potency similar to Cry2. The abscissa indicates the day in culture, and the ordinate the relative bioluminescence intensity (kcpm, 1,000 photon counts per minute). The first amplitude (D) was significantly decreased with overexpression of Chrono compared to control. The bar plots indicate the mean ± S.E.M (shaded area) of eight samples. **p<0.0001, Student's t test. (E) Dec2 KO MEFs were transfected with combinations of expression vectors as indicated. Chrono inhibited BMAL1–CLOCK complex-induced transcriptional activity on the mPer2 promoter. The effects of overexpression of BMAL1, CLOCK, DEC2, and CHRONO proteins on Per2 transcription were evaluated by measuring bioluminescence from luciferase activities. The basal transcription level of the Per2 promoter was set to 1. The bar plots indicate the mean ± S.E.M of triplicate samples. *p<0.001, Student's t test. (F) ChIP analyses for HDAC1 and IgG (negative control). The HDAC1 occupancies at the endogenous E-box of the Per2 promoter were detected in the WT MEF cells at 28, 36, 44, and 52 h after induction with dexamethasone. The graph showed relative real-time PCR values. The maximum value of WT was set to 100. Data are means ± S.E.M. of three samples. (F) ChIP analysis for BMAL1 an [file pbio.1001839.s003.tif]

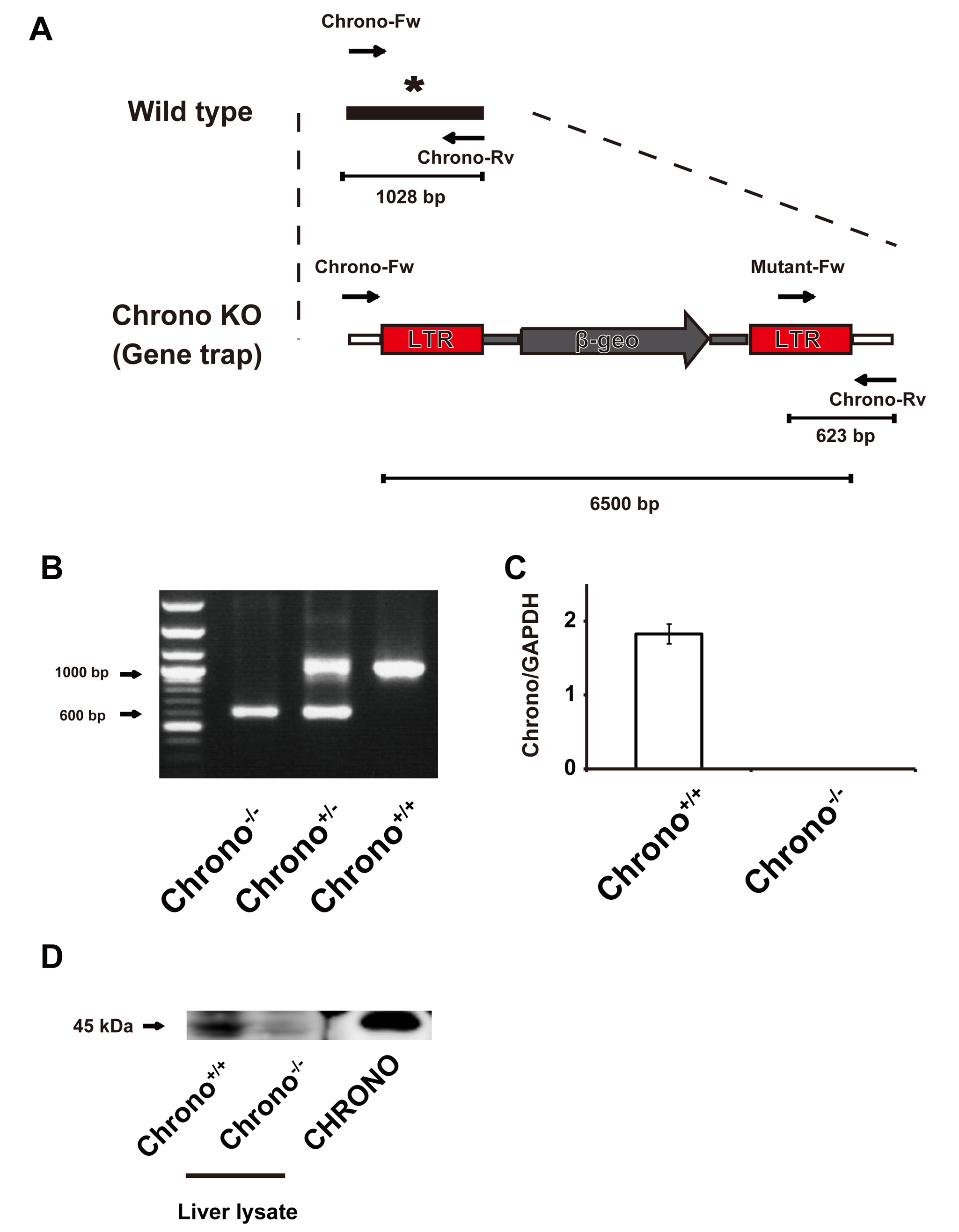

Supplement: Figure S4 — Construction of Chrono KO mice. (A) The targeting strategy for PCR genotyping. An LTR–splice acceptor–βgeo–polyA–LTR sequence is inserted between exons 1 and 2 of Chrono allele (TIGM, Texas A&M Institute for Genomic Medicine). Primer locations are schematically displayed in (A). (B) PCR genotyping of DNA extracted from mouse tails of KO (Chrono −/−), heterozygous (Chrono +/−), WT (Chrono +/+) offspring. (C) RT-PCR analysis of Chrono expression in the hypothalamus. (D) Western blot analysis of Chrono expression in the liver of KO (Chrono −/−) and WT (Chrono +/+). (TIF) [file pbio.1001839.s004.tif]

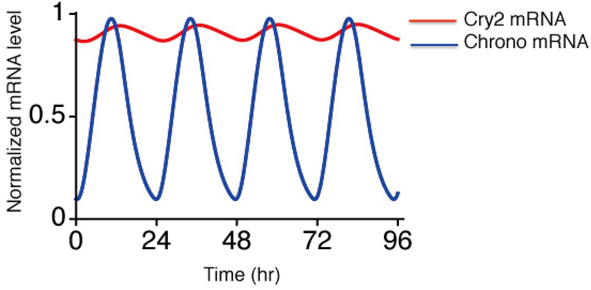

Supplement: Figure S5 — The simulated time courses of Chrono mRNA and Cry2 mRNA in the mathematical model. The amplitude and phase of Chrono mRNA and Cry2 mRNA are very different, matching experimental data [22],[47]. That is, the amplitude of the Chrono mRNA rhythm is much larger than that of Cry2 mRNA, and the phase of the Chrono mRNA rhythm is more advanced than that of Cry2 mRNA. (TIF) [file pbio.1001839.s005.tif]

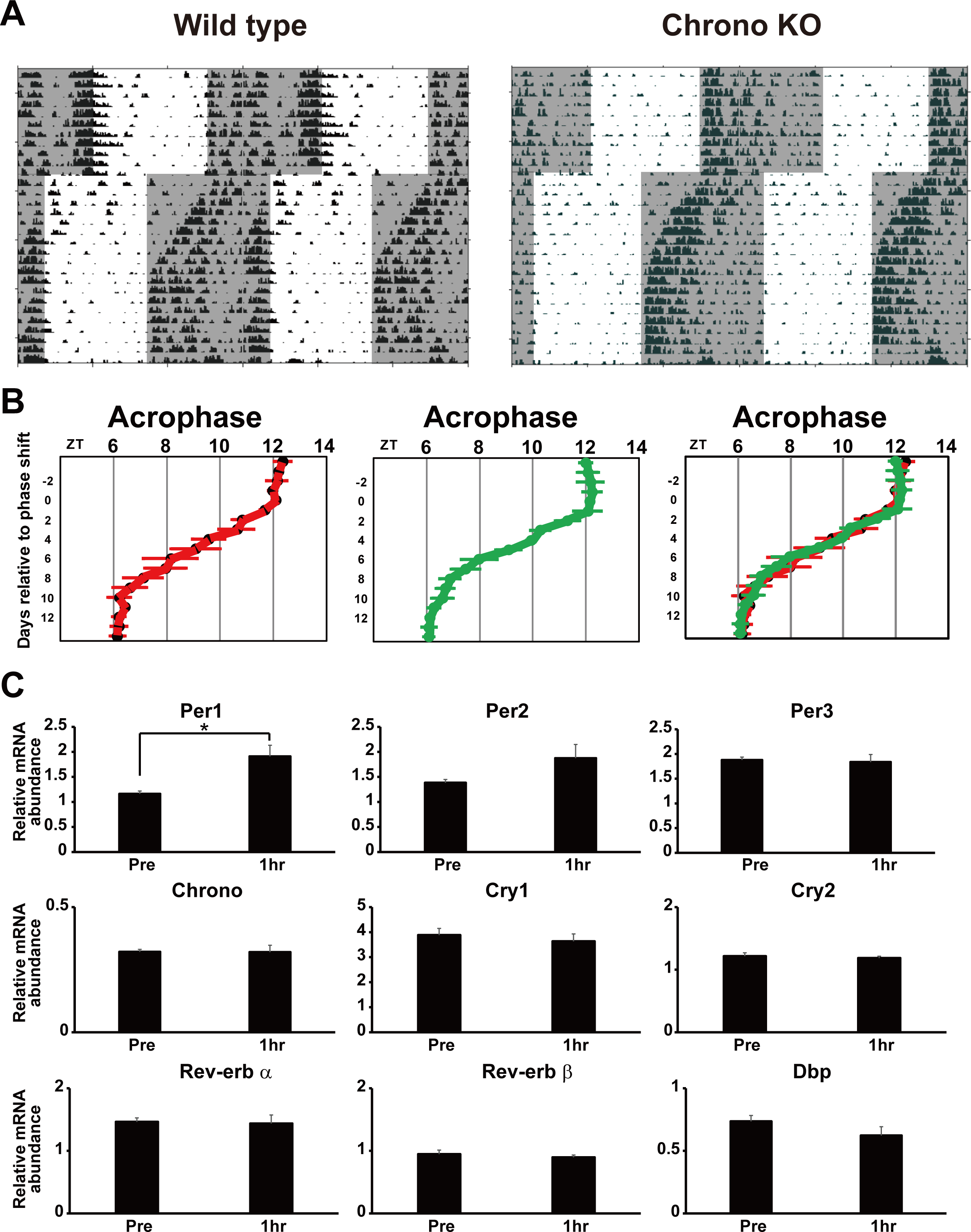

Supplement: Figure S6 — Characterization of Chrono KO and light response of Chrono in vivo . (A) Representative actograms from WT and Chrono KO mice that were subjected first to LD cycles, followed by a 6-h jet-lag light phase advance. Shaded areas indicate the dark phase. (B) Re-entrainment traces from an average of WT (red), Chrono KO (green), and merged (right). (C) Although a 30 min light pulse (1,000 lux) delivered from CT16.0 to CT16.5 induced Per1 mRNA expression (*p<0.05, Student's t test), Chrono expression was not induced. (TIF) [file pbio.1001839.s006.tif]

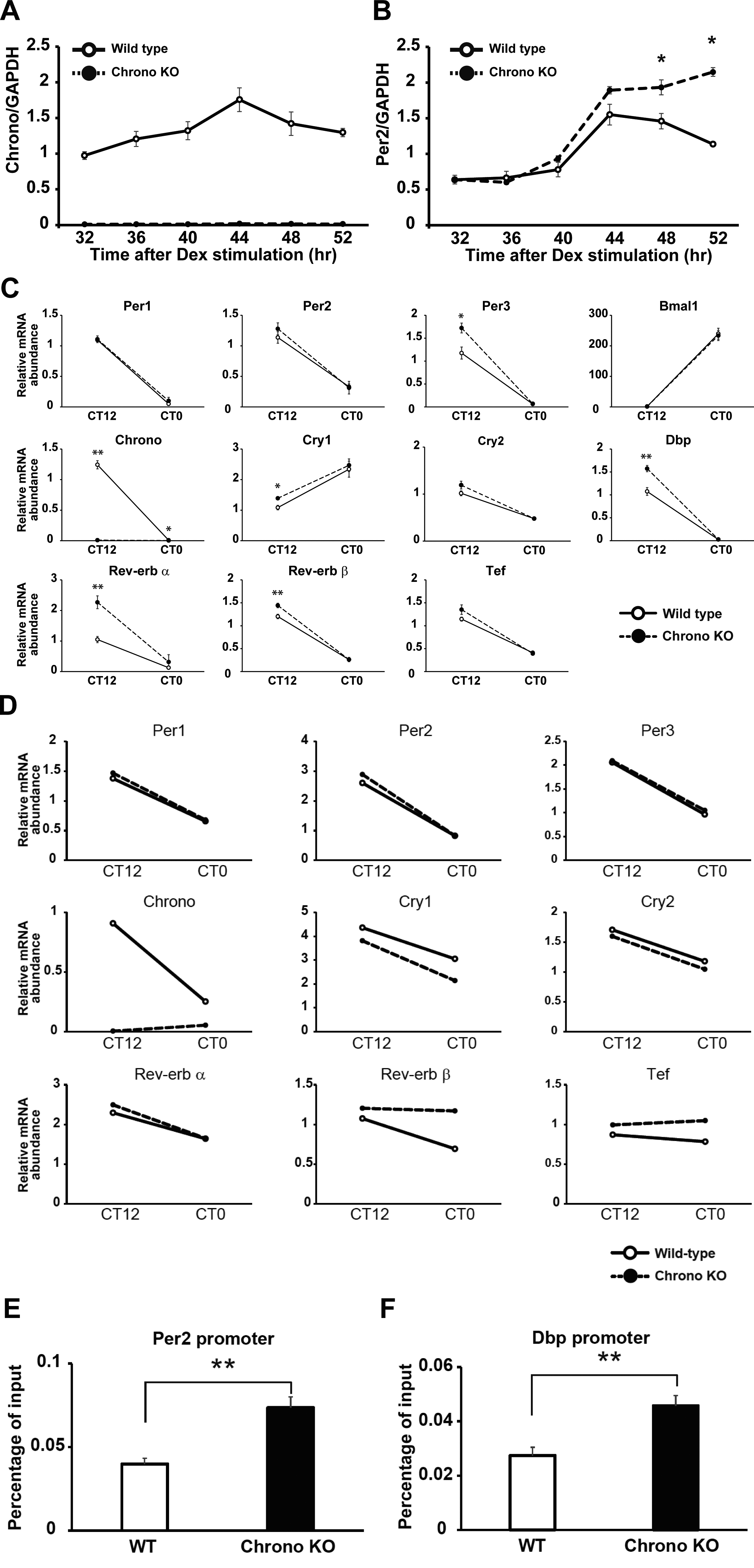

Supplement: Figure S7 — Characterization of Chrono KO and Chrono may change the epigenetic modification of cells via Acetyl-Histone H3. Analysis of mRNA of core clock genes in WT and Chrono KO MEFs. Temporal mRNA expression of Chrono (A) and Per2 (B) in WT and Chrono KO MEFs. The abscissa represents time after dexamethasone stimulation and the ordinate the mRNA amounts. (C) mRNA expressions of circadian genes in WT and Chrono KO liver at CT12 and CT0. The relative levels of mRNA were normalized to the corresponding GAPDH mRNA levels. Plots and error bars represent mean ± S.E.M. of four samples. *p<0.05, **p<0.01, Student's t test. (D) Expression patterns of circadian genes in the SCN of WT and Chrono KO mice. The SCN samples from four or five mice were mixed at each time point. Solid lines with white circles and dotted lines with black circles represent WT and Chrono KO, respectively. The relative levels of each mRNA are normalized to the corresponding GAPDH RNA level. (E and F) ChIP analysis for Acetyl-Histone H3. The Acetyl-Histone H3 occupancies at the endogenous E-boxes of Per2 (E) and Dbp (F) promoters were detected in WT and Chrono KO MEFs at 52 h after induction with dexamethasone. The data were plotted as percentages relative to the input DNA. Data are means ± S.E.M. of five samples. (TIF) [file pbio.1001839.s007.tif]

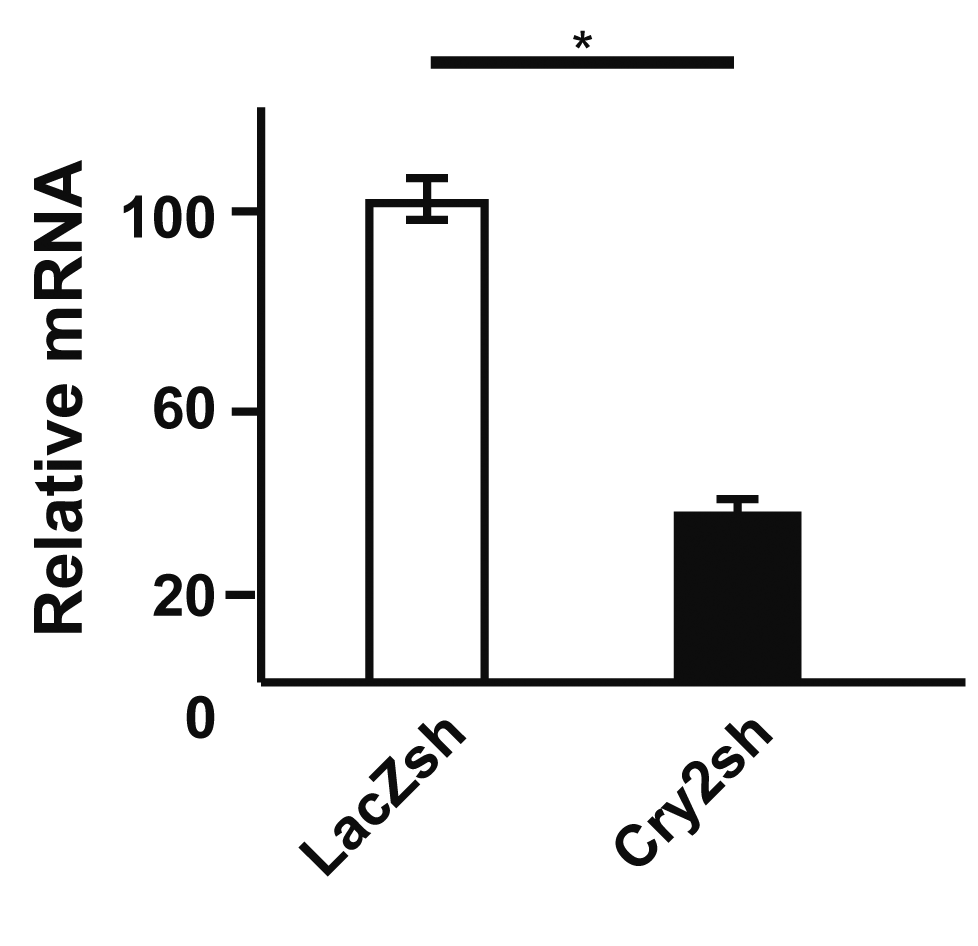

Supplement: Figure S8 — RT-PCR quantification of Cry2 mRNA abundance in NIH3T3 fibroblasts that stably express the Cry2 shRNA. LacZsh was used as a control. The relative levels of mRNA were normalized to the corresponding GAPDH mRNA levels. The maximum mRNA amount was set to 100. *p<0.01, Student's t test. (TIF) [file pbio.1001839.s008.tif]

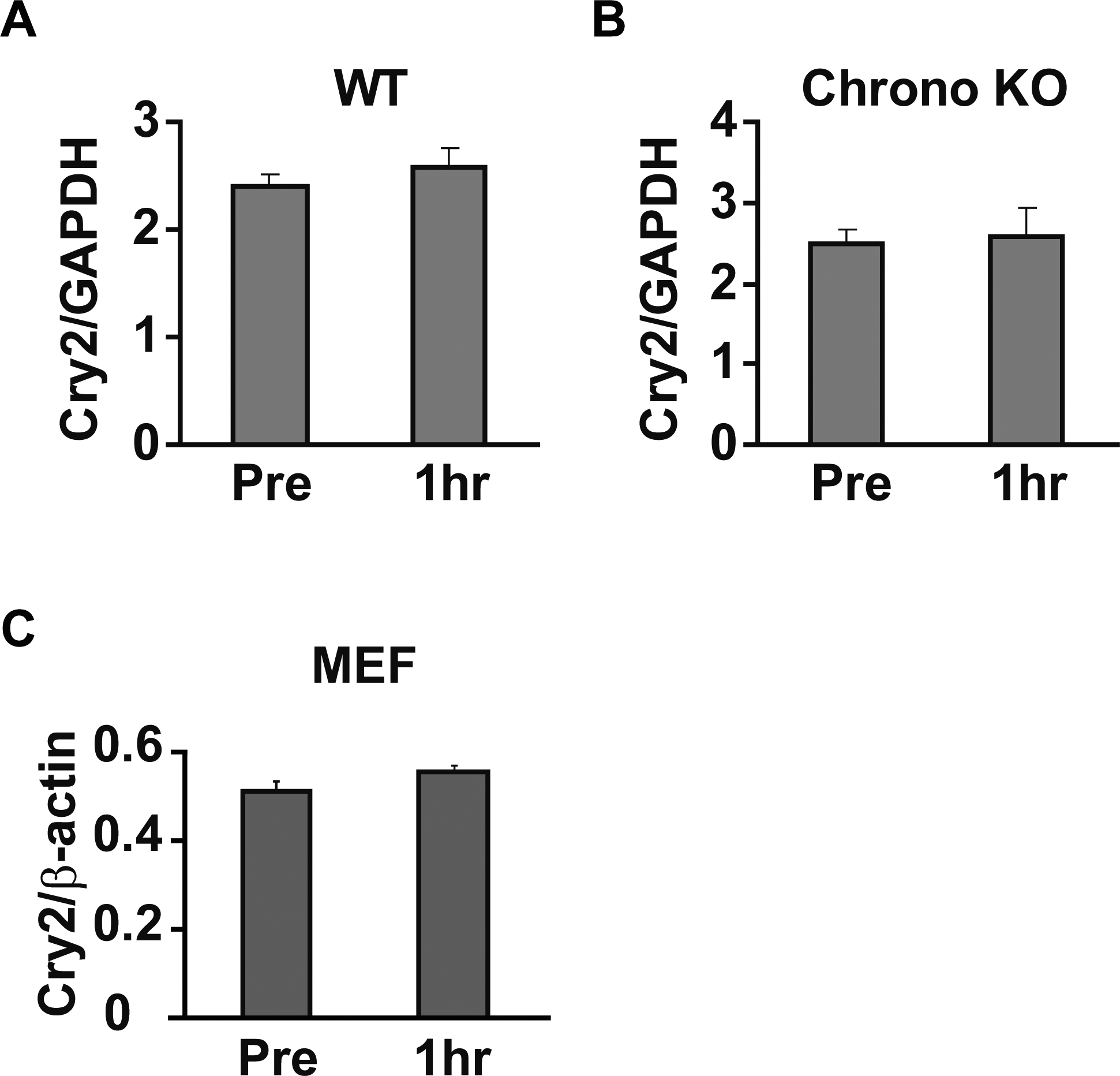

Supplement: Figure S9 — Expression of Cry2 in hypothalamus under restraint stress. Expression of Cry2 in hypothalamus under restraint stress (pre- and after 1 h) of WT (A) and Chrono KO mice (B). The relative level of Cry2 mRNA was normalized to the corresponding GAPDH mRNA. Expression of Cry2 in WT MEFs after Dex stimulation (C) (pre- and after 1 h). (TIF) [file pbio.1001839.s009.tif]

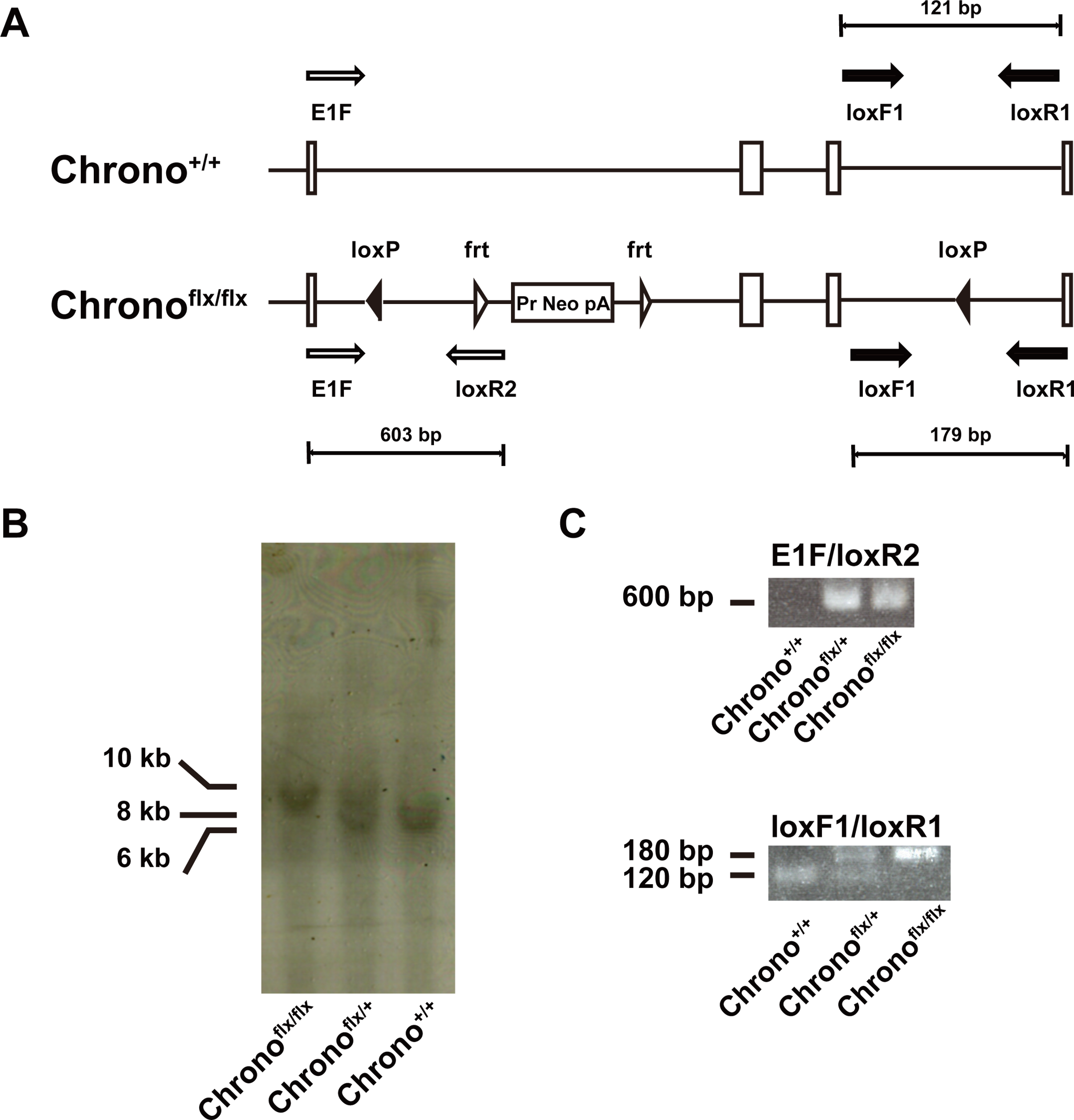

Supplement: Figure S10 — Construction of Avp -specific KO of Chrono in mice. (A) The targeting strategy for PCR genotyping illustrated by the structures of the WT Chrono allele and the floxed allele Chrono flx. Chrono exons are represented as open boxes and 5′ genomic DNA, intronic sequences, and 3′ genomic DNA as solid lines. The Pr Neo pA and the Pr DT-A pA cassettes are shown as open boxes. The loxP sequences are indicated as solid triangles and the frt sequences as open triangles. (B) Southern blot verification of mouse tail DNA containing the Chrono flx allele. Extracted DNA samples were digested with AflIII and hybridized with the probe (Figure 7A). The WT (6.7 kb) and Chrono flx (8.7 kb) alleles were detected. (C) Mouse tail DNA from mice carrying WT and/or the Chronoflx allele were genotyped by PCR using primer sets E1F/loxR2 or loxF1/loxR1, as shown in (A). E1F/loxR2 amplifies a fragment of 603 bp only from the Chrono flx allele. loxF1/loxR1 amplifies fragments of 121 bp from the WT allele and 179 bp from the Chrono flx allele. (TIF) [file pbio.1001839.s010.tif]

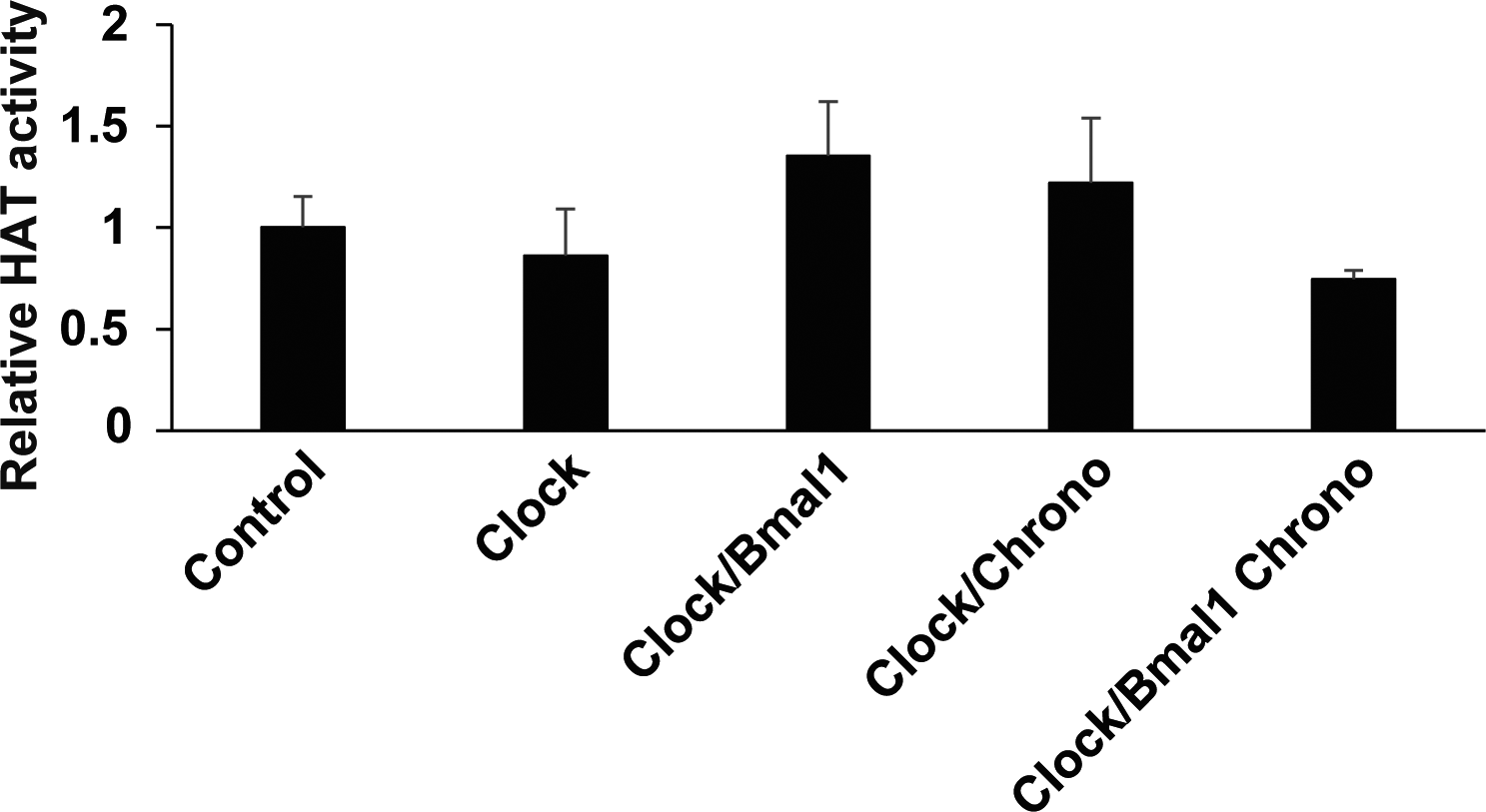

Supplement: Figure S11 — HAT activity in NIH3T3. HAT activity in NIH3T3 cells, which were transfected with the desired plasmids by using Lipofectamine 2000 (Invitrogen). After 24 h from transfection, the nuclear protein was extracted and the Histone acetyltransferase activity was measured by using HAT assay kits (ab65352, Abcam). (TIF) [file pbio.1001839.s011.tif]
