# Supplementary material for: A Novel Protein, CHRONO, Functions as a Core Component of the Mammalian Circadian Clock
Source: PLoS Biol. 2014 Apr 15;12(4):e1001839. doi: 10.1371/journal.pbio.1001839 (PMC3988004; doi:10.1371/journal.pbio.1001839)
Supplement: Table S1 — Newly added variables for mRNA dynamics of Chrono . (DOCX) [file pbio.1001839.s012.docx]

**Supplementary Table 1.** Newly added variables for mRNA dynamics of *Chrono*.

| **Name** | **Symbol** |
| --- | --- |
| The concentration of *Chrono* mRNA in the nucleus | MnCh |
| The concentration of *Chrono* mRNA in the cytoplasm | McCh |

The rest of single-state variables can be found in Table S1 in Kim and Forger (2012).
