# Supplementary material for: A Novel Protein, CHRONO, Functions as a Core Component of the Mammalian Circadian Clock
Source: PLoS Biol. 2014 Apr 15;12(4):e1001839. doi: 10.1371/journal.pbio.1001839 (PMC3988004; doi:10.1371/journal.pbio.1001839)
Supplement: Table S2 — Extended variables of protein complexes. (DOCX) [file pbio.1001839.s013.docx]

**Supplementary Table 2.** Extended variables of protein complexes.

| **Index** | ***j***  **PER** | ***k***  **CRY** | ***l***  **Kinase** | ***m***  **Location** | ***n***  **BMALs-CLK** |
| --- | --- | --- | --- | --- | --- |
| **0** | No PER bound | No CRY bound | No Kinases bound | Cytoplasm | No BMALs-CLK bound |
| **1** | PER1 | CRY1 | CKI | Nucleus | BMALs^P^-CLK^P^ |
| **2** | PER1^P^ by CKI | CRY2 | GSK3 |  |  |
| **3** | PER2 | CHRONO | CKI&GSK3 |  |  |
| **4** | PER2^p^ by CKI |  |  |  |  |
| **5** | PER2^P^ by GSK3 |  |  |  |  |
| **6** | PER2^P^ by both GSK3 and CKI |  |  |  |  |

Each complex is denoted x[*j*][*k*][*l*][*m*][*n*], where *j*, *k*, *l*, *m*, and *n* refer to the proteins that are present in the complex, or the location of the complex. See the Supplementary Table S2 in the original model paper for the details of the notations (Kim and Forger, 2012).
