# Supplementary material for: A Novel Protein, CHRONO, Functions as a Core Component of the Mammalian Circadian Clock
Source: PLoS Biol. 2014 Apr 15;12(4):e1001839. doi: 10.1371/journal.pbio.1001839 (PMC3988004; doi:10.1371/journal.pbio.1001839)
Supplement: Table S3 — Newly added/modified parameters for Chrono dynamics. (DOCX) [file pbio.1001839.s014.docx]

**Supplementary Table 3.** Newly added/modified parameters for *Chrono* dynamics.

| **Parameter description** | **Symbol** | **Value** |
| --- | --- | --- |
| Transcription rate constant for *Per2/Chrono* | trPt | 25.92/hr |
| Translation rate constant for CHRONO | Tlch | 2.2/hr |
| Binding rate constant for PER2 to CRY1/CRY2/CHRONO | ar | 0.024/nM hr |
| Unbinding rate constant for PER2 to CRY1/CRY2/CHRONO | dr | 0.605/hr |
| Binding rate constant for CRY1/CRY2/CHRONO to BMAL-CLOCK/NPAS2 in the nucleus | cbbin | 6.599/nM hr |
| Unbinding rate constant for CRY1/CRY2/CHRONO to BMAL-CLOCK/NPAS2 in the nucleus | uncbbin | 0.304/hr |
| Normalized binding rate constant for BMAL-CLOCK/NPAS2 to *Per1/2/Cry1/Chrono* E-box | bin | 6.972/hr |
| Normalized unbinding rate constant for BMAL-CLOCK/NPAS2 to *Per1/2/Cry1/Chrono* E-box | unbin | 0.255/hr |
| Rate constant for folding and nuclear export of *Per1/2, Cry1/2, Bmal*, *Npas2,* and *Chrono* mRNA | tmc | 0.164/hr |
| Degradation rate constant for *Per2*/Chrono mRNA | umPt | 0.589/hr |
| Degradation rate constant for CRY2/CHRONO protein | urt | 0.482/hr |
| Modified factor for transcription rate constant for *Per1*/*Per2*/*Cry1*/*Cry2*/*Rev-erbs* | vch | 1.1 |

See Supplementary Table 3 in the original model paper for the rest of parameters (Kim and Forger, 2012).
