# Supplementary material for: A Novel Protein, CHRONO, Functions as a Core Component of the Mammalian Circadian Clock
Source: PLoS Biol. 2014 Apr 15;12(4):e1001839. doi: 10.1371/journal.pbio.1001839 (PMC3988004; doi:10.1371/journal.pbio.1001839)
Supplement: Text S1 — Supplementary methods. (DOCX) [file pbio.1001839.s015.docx]

**Supplementary Methods**

**Extension of the Kim-Forger model for *Chrono* dynamics**

We incorporated *Chrono* into a recently developed mathematical model of the intracellular mammalian circadian clock (Kim and Forger, 2012). Since our experimental data indicated that *Cry2* and *Chrono* have similar biochemical properties, we assumed that they were the same in the model except for the following:

*Transcription of Chrono:* Since we showed that *Chrono* has E-boxes in its promoter, transcription of *Chrono* mRNA should be proportional to the activity of E-boxes on the *Chrono* promoter. We further assumed that the E-boxes of *Chrono* and *Per2* promoters are under the same dynamical control and the degradation rates of *Chrono* and *Per2* mRNA are the same as *Chrono* mRNA exhibited a similar time course to that of *Per2* mRNA (Figure 1B; Yamamoto et al., 2005). As a result, in the model simulation, the amplitude of the *Chrono* mRNA rhythm is much larger than that of *Cry2* mRNA rhythm, and the phase of the *Chrono* mRNA rhythm is more advanced than that of *Cry2* mRNA rhythm (Figure S5) (Hatanaka *et al.* (2010) and Ueda *et al.* (2005)).

*Repression of BMAL1-CLOCK by CHRONO:* *Chrono* mRNA is translated to CHRONO protein in the cytoplasm. Whereas CRY2 protein binds both PER1/2, CHRONO protein binds to PER2 protein, but not to PER1 as shown in our experiments (Figure 2A). The complex of PER2-CHRONO is assumed to enter the nucleus in a manner similar to PER1/2-CRY1/2. The CHRONO protein in the nucleus binds to BMAL1-CLOCK and inhibits the binding of BMAL1-CLOCK to E-boxes in *Per1*, *Per2*, *Cry1*, *Cry2*, and *Rev-erb*s promoters, matching our experimental data (Figure 2A and B). In the model, we did not consider binding between CHRONO and CRY2 since our experiments indicated that BMAL1-CLOCK repression by CHRONO did not depend on the presence of CRY2 (Figure 5). The novel introduction of the *Chrono* variables into the model added extra suppression of the E-box activity in the promoters of *Per1*, *Per2*, *Cry1*, *Cry2* and *Rev-erb*s compared to the original model. To compensate for the suppression, the transcription rates of *Per1*, *Per2*, *Cry1*, *Cry2* and *Rev-erb*s were increased by 10%.

**Newly added variables and parameters in the mathematical model**

To describe the dynamics of *Chrono*, 38 variables were newly added: 2 for mRNA of *Chrono* (Table S1) and 36 for protein complexes of CHRONO with other proteins, such as PER2 and BMAL1-CLOCK (Table S2). See the original model paper for the details of the notation of the variables in the model (Kim and Forger, 2012).

Twelve parameters were needed to describe the dynamics of *Chrono* in the model (Table S3). Most of the parameters were adopted from the parameters used in the original model following our assumptions: similarity of transcription of *Per2* and *Chrono* and similarity of CRY2 and CHRONO protein. Only the translation rate of *Chrono* was not taken from the parameters in the original Kim-Forger model. The choice of translation rate of *Chrono* does not change the quantitative behavior model, such longer period in *Chrono* KO (Figure 4). The translation of *Chrono* mRNA was selected that reproduce the period of *Chrono* KO phenotype quantitatively (Figure 5G).

**Computer simulation of the model**

All the simulations were performed with Mathematica 8.0 (Wolfram Research).

**Appendix**

**Newly added and modified equations:** These equations follow the format and conventions of the original models

**1) Transcription of Chrono**

MnCh'[t]=trPt*G[t]-tmc*MnCh[t]-umPt*MnCh[t]

McCh'[t]=tmc*MnCh[t]-umPt*McCh[t]

**2) Increased transcription rates of Per1, Per2, Cry1, Cry2 and Rev-erbs**

MnPo'[t]==vch*trPo*G[t]-tmc*MnPo[t]-umPo*MnPo[t],

MnPt'[t]==vch*trPt*G[t]-tmc*MnPt[t]-umPt*MnPt[t],

MnRt'[t]==vch*trRt*Gc[t]-tmc*MnRt[t]-umRt*MnRt[t],

MnRev'[t]==vch*trRev*x[0][0][0][1][1][t]*Gr[t]-tmcrev*MnRev[t]-umRev*MnRev[t],

MnRo'[t]==vch*trRo*G[t]*GB[t]-tmc*MnRo[t]-umRo*MnRo[t]

**3) Translation of Chrono**

x[j][k][l][m][n]'=If[(j==0)&&(k==3)&&(l==0)&&(m==0)&&(n==0),tlCh*McCh[t],0]

**4) Binding and unbinding between CHRONO and PER2**

x[j][k][l][m][n]'=

If[(k==0)&&(n==0)&&(j>3),-ar*If[m==1,Nf,1]*x[0][3][0][m][0][t]*x[j][k][l][m][n][t]+dr*x[j][3][l][m][n][t],0]+

If[(j==0)&&(k==3)&&(l==0)&&(n==0),-ar*If[m==1,Nf,1]* x[j][k][l][m][n][t]* Sum[x[jj][0][ll][m][0][t],{jj,{4,5,6}},{ll,0,3}]+dr*Sum[x[jj][k][ll][m][n][t],{jj,{4,5,6}},{ll,0,3}],0]+

If[(j>3)&&(k==3)&&(n==0), ar*If[m==1,Nf,1]*x[0][k][0][m][n][t]*x[j][0][l][m][0][t]- dr*x[j][k][l][m][n][t],0]+

If[(k==0)&&(n==1)&&(j>3)&&(m== 1),-ar*Nf*x[j][k][l][m][n][t]*x[0][3][0][m][0][t]+dr*x[j][3][l][m][n][t],0]+

If[(j==0)&&(k==3)&&(l==0)&&(m==1)&&(n==0),-ar*Nf* x[j][k][l][m][n][t]* Sum[x[jj][0][ll][m][1][t],{jj,{4,5,6}},{ll,0,3}]+ dr*Sum[x[jj][k][ll][m][1][t],{jj,{4,5,6}},{ll,0,3}],0]+

If[(j>3)&&(k==3)&&(m==1)&&(n==1), ar*Nf*x[j][0][l][m][n][t]*x[0][k][0][m][0][t]-dr*x[j][k][l][m][n][t], 0]+

If[(k==0)&&(n==0)&&(j>3)&&(m== 1),-ar*Nf*x[j][k][l][m][n][t]*x[0][3][0][m][1][t]+dr*x[j][3][l][m][1][t],0]+

If[(j==0)&&(k==3)&&(l==0)&&(m==1)&&(n==1),-ar*Nf* x[j][k][l][m][n][t]* Sum[x[jj][0][ll][m][0][t],{jj,{4,5,6}},{ll,0,3}]+dr*Sum[x[jj][k][ll][m][n][t],{jj,{4,5,6}},{ll,0,3}],0]+

If[(j>3)&&(k==3)&&(m==1)&&(n==1),ar*Nf*x[j][0][l][m][0][t]*x[0][k][0][m][1][t]-dr*x[j][k][l][m][n][t],0]

**4) Binding and unbinding between CHRONO-PER2 and CK1**

x[j][k][l][m][n]'=

If[(l==0)&&(j>0)&&(n==0),-ac*If[m==1,Nf,1]*x[j][k][l][m][n][t]* x[0][0][1][m][0][t]+dc*x[j][k][1][m][n][t],0]+

If[(j==0)&&(k==0)&&(l==1)&&(n==0),-ac*If[m==1,Nf,1]* x[j][k][l][m][n][t]*Sum[x[jj][kk][0][m][0][t],{jj,1,6},{kk,0,3}]+dc*Sum[x[jj][kk][l][m][0][t],{jj,1,6},{kk,0,3}],0]+

If[(j>0)&&(l==1)&&(n==0), ac*If[m==1,Nf,1]*x[0][0][1][m][0][t]*x[j][k][0][m][n][t]-dc*x[j][k][l][m][n][t],0]

+If[(l==0)&&(j>0)&&(m==1)&&(n==1),-ac*Nf*x[j][k][l][m][n][t]* x[0][0][1][m][0][t]+dc*x[j][k][1][m][n][t],0]+

If[(j==0)&&(k==0)&&(l==1)&&(m==1)&&(n==0),-ac*Nf* x[j][k][l][m][n][t]*Sum[x[jj][kk][0][m][1][t],{jj,1,6},{kk,0,3}]+ dc*Sum[x[jj][kk][l][m][1][t],{jj,1,6},{kk,0,3}],0]+

If[(j>0)&&(l==1)&&(m==1)&&(n==1), ac*Nf*x[0][0][1][m][0][t]*x[j][k][0][m][n][t]-dc*x[j][k][l][m][n][t],0]+

If[(j>2)&&(l==2)&&(n==0),-ac*If[m==1,Nf,1]* x[j][k][l][m][n][t]*x[0][0][1][m][0][t]+dc*x[j][k][3][m][n][t],0]+

If[(j==0)&&(k==0)&&(l==1)&&(n==0),-ac*If[m==1,Nf,1]* x[j][k][l][m][n][t]*Sum[x[jj][kk][2][m][0][t],{jj,3,6},{kk,0,3}]+ dc*Sum[x[jj][kk][3][m][0][t],{jj,3,6},{kk,0,3}],0]+

If[(j>2)&&(l==3)&&(n==0), ac*If[m==1,Nf,1]*x[0][0][1][m][0][t]*x[j][k][2][m][n][t]-dc*x[j][k][l][m][n][t],0]+

If[(j>2)&&(l==2)&&(m==1)&&(n==1),-ac*Nf*x[j][k][l][m][n][t]* x[0][0][1][m][0][t]+dc*x[j][k][3][m][n][t],0]+

If[(j==0)&&(k==0)&&(l==1)&&(m==1)&&(n==0),-ac*Nf* x[j][k][l][m][n][t]*Sum[x[jj][kk][2][m][1][t],{jj,3,6},{kk,0,3}]+ dc*Sum[x[jj][kk][3][m][1][t],{jj,3,6},{kk,0,3}],0]+

If[(j>2)&&(l==3)&&(m==1)&&(n==1), ac*Nf*x[0][0][1][m][0][t]*x[j][k][2][m][n][t]-dc*x[j][k][l][m][n][t], 0]

**5) Binding and unbinding between CHRONO-PER2 and GSK3b**

x[j][k][l][m][n]'=

If[(j>2)&&((l==0)||(l==1)),-If[m==1,Nf,1]*agp* x[j][k][l][m][n][t]*x[0][0][2][m][0][t]+dg*x[j][k][l+2][m][n][t],0]+

If[(j==0)&&(k==0)&&(l==2)&&(n==0),-If[m==1,Nf,1]*agp* Sum[x[jj][kk][ll][m][nn][t],{jj,3,6},{kk,0,3},{ll,0,1},{nn,0, 1}]*x[j][k][l][m][n][t]+ dg*Sum[ x[jj][kk][ll][m][nn][t],{jj,3,6},{kk,0,3},{ll,2,3},{nn,0,1}], 0]+

If[(j>2)&&((l==2)||(l==3)), If[m==1,Nf,1]*agp*x[j][k][l-2][m][n][t]*x[0][0][2][m][0][t]- dg*x[j][k][l][m][n][t],0]

**6) Binding and unbinding between CHRONO and BMAL1-CLOCK**

x[j][k][l][m][n]'=

If[(j==0)&&(k>0)&&(l==0)&&(m==1)&&(n==0),-cbbin*Nf* x[j][k][l][m][n][t]*x[0][0][0][m][1][t]+uncbbin*x[j][k][l][m][1][t], 0]+

If[(j==0)&&(k==0)&&(l==0)&&(m==1)&&(n==1),-cbbin*Nf* Sum[x[0][kk][0][m][0][t],{kk,1,3}]*x[j][k][l][m][n][t]+ uncbbin*Sum[x[0][kk][0][m][n][t],{kk,1,3}],0]+

If[(j==0)&&(k>0)&&(l==0)&&(m==1)&&(n==1),cbbin*Nf*x[j][k][l][m][0][t]*x[0][0][0][m][n][t]- uncbbin*x[j][k][l][m][n][t],0]

**7) PER2-CHRONO subcellular translocation**

x[j][k][l][m][n]'=

If[((j==2)||(j==4)||(j==5)||(j==6))&&(m==1),-ne* If[(n==0),1,0]*x[j][k][l][m][n][t]+If[(n==0),1,0]*nl*x[j][k][l][0][n][t],0]+

If[((j==2)||(j==4)||(j==5)||(j==6))&&(m==0), ne*If[(n==0),1,0]*x[j][k][l][1][n][t]-If[(n==0),1,0]*nl*x[j][k][l][m][n][t],0]

**8) CHRONO degradation**

x[j][k][l][m][n]'=

If[(j==0)&&(k==3)&&(l==0)&&(n==0),-urt*x[j][k][l][m][n][t], 0]+

If[(j==0)&&(k==3)&&(l==0)&&(m==1)&&(n==1),-urt* x[j][k][l][m][n][t],0]+

If[(j==0)&&(k==0)&&(l==0)&&(m==1)&&(n==1),urt*x[j][3][l][m][n][t], 0]
