# Supplementary material for: A Novel Protein, CHRONO, Functions as a Core Component of the Mammalian Circadian Clock
Source: PLoS Biol. 2014 Apr 15;12(4):e1001839. doi: 10.1371/journal.pbio.1001839 (PMC3988004; doi:10.1371/journal.pbio.1001839)
Supplement: Appendix S1 — Newly added and modified equations. (DOCX) [file pbio.1001839.s016.docx]

**Appendix**

**Newly added and modified equations:** These equations follow the format and conventions of the original models

**1) Transcription of Chrono**

MnCh'[t]=trPt*G[t]-tmc*MnCh[t]-umPt*MnCh[t]

McCh'[t]=tmc*MnCh[t]-umPt*McCh[t]

**2) Increased transcription rates of Per1, Per2, Cry1, Cry2 and Rev-erbs**

MnPo'[t]==vch*trPo*G[t]-tmc*MnPo[t]-umPo*MnPo[t],

MnPt'[t]==vch*trPt*G[t]-tmc*MnPt[t]-umPt*MnPt[t],

MnRt'[t]==vch*trRt*Gc[t]-tmc*MnRt[t]-umRt*MnRt[t],

MnRev'[t]==vch*trRev*x[0][0][0][1][1][t]*Gr[t]-tmcrev*MnRev[t]-umRev*MnRev[t],

MnRo'[t]==vch*trRo*G[t]*GB[t]-tmc*MnRo[t]-umRo*MnRo[t]

**3) Translation of Chrono**

x[j][k][l][m][n]'=If[(j==0)&&(k==3)&&(l==0)&&(m==0)&&(n==0),tlCh*McCh[t],0]

**4) Binding and unbinding between CHRONO and PER2**

x[j][k][l][m][n]'=

If[(k==0)&&(n==0)&&(j>3),-ar*If[m==1,Nf,1]*x[0][3][0][m][0][t]*x[j][k][l][m][n][t]+dr*x[j][3][l][m][n][t],0]+

If[(j==0)&&(k==3)&&(l==0)&&(n==0),-ar*If[m==1,Nf,1]* x[j][k][l][m][n][t]* Sum[x[jj][0][ll][m][0][t],{jj,{4,5,6}},{ll,0,3}]+dr*Sum[x[jj][k][ll][m][n][t],{jj,{4,5,6}},{ll,0,3}],0]+

If[(j>3)&&(k==3)&&(n==0), ar*If[m==1,Nf,1]*x[0][k][0][m][n][t]*x[j][0][l][m][0][t]- dr*x[j][k][l][m][n][t],0]+

If[(k==0)&&(n==1)&&(j>3)&&(m== 1),-ar*Nf*x[j][k][l][m][n][t]*x[0][3][0][m][0][t]+dr*x[j][3][l][m][n][t],0]+

If[(j==0)&&(k==3)&&(l==0)&&(m==1)&&(n==0),-ar*Nf* x[j][k][l][m][n][t]* Sum[x[jj][0][ll][m][1][t],{jj,{4,5,6}},{ll,0,3}]+ dr*Sum[x[jj][k][ll][m][1][t],{jj,{4,5,6}},{ll,0,3}],0]+

If[(j>3)&&(k==3)&&(m==1)&&(n==1), ar*Nf*x[j][0][l][m][n][t]*x[0][k][0][m][0][t]-dr*x[j][k][l][m][n][t], 0]+

If[(k==0)&&(n==0)&&(j>3)&&(m== 1),-ar*Nf*x[j][k][l][m][n][t]*x[0][3][0][m][1][t]+dr*x[j][3][l][m][1][t],0]+

If[(j==0)&&(k==3)&&(l==0)&&(m==1)&&(n==1),-ar*Nf* x[j][k][l][m][n][t]* Sum[x[jj][0][ll][m][0][t],{jj,{4,5,6}},{ll,0,3}]+dr*Sum[x[jj][k][ll][m][n][t],{jj,{4,5,6}},{ll,0,3}],0]+

If[(j>3)&&(k==3)&&(m==1)&&(n==1),ar*Nf*x[j][0][l][m][0][t]*x[0][k][0][m][1][t]-dr*x[j][k][l][m][n][t],0]

**4) Binding and unbinding between CHRONO-PER2 and CK1**

x[j][k][l][m][n]'=

If[(l==0)&&(j>0)&&(n==0),-ac*If[m==1,Nf,1]*x[j][k][l][m][n][t]* x[0][0][1][m][0][t]+dc*x[j][k][1][m][n][t],0]+

If[(j==0)&&(k==0)&&(l==1)&&(n==0),-ac*If[m==1,Nf,1]* x[j][k][l][m][n][t]*Sum[x[jj][kk][0][m][0][t],{jj,1,6},{kk,0,3}]+dc*Sum[x[jj][kk][l][m][0][t],{jj,1,6},{kk,0,3}],0]+

If[(j>0)&&(l==1)&&(n==0), ac*If[m==1,Nf,1]*x[0][0][1][m][0][t]*x[j][k][0][m][n][t]-dc*x[j][k][l][m][n][t],0]

+If[(l==0)&&(j>0)&&(m==1)&&(n==1),-ac*Nf*x[j][k][l][m][n][t]* x[0][0][1][m][0][t]+dc*x[j][k][1][m][n][t],0]+

If[(j==0)&&(k==0)&&(l==1)&&(m==1)&&(n==0),-ac*Nf* x[j][k][l][m][n][t]*Sum[x[jj][kk][0][m][1][t],{jj,1,6},{kk,0,3}]+ dc*Sum[x[jj][kk][l][m][1][t],{jj,1,6},{kk,0,3}],0]+

If[(j>0)&&(l==1)&&(m==1)&&(n==1), ac*Nf*x[0][0][1][m][0][t]*x[j][k][0][m][n][t]-dc*x[j][k][l][m][n][t],0]+

If[(j>2)&&(l==2)&&(n==0),-ac*If[m==1,Nf,1]* x[j][k][l][m][n][t]*x[0][0][1][m][0][t]+dc*x[j][k][3][m][n][t],0]+

If[(j==0)&&(k==0)&&(l==1)&&(n==0),-ac*If[m==1,Nf,1]* x[j][k][l][m][n][t]*Sum[x[jj][kk][2][m][0][t],{jj,3,6},{kk,0,3}]+ dc*Sum[x[jj][kk][3][m][0][t],{jj,3,6},{kk,0,3}],0]+

If[(j>2)&&(l==3)&&(n==0), ac*If[m==1,Nf,1]*x[0][0][1][m][0][t]*x[j][k][2][m][n][t]-dc*x[j][k][l][m][n][t],0]+

If[(j>2)&&(l==2)&&(m==1)&&(n==1),-ac*Nf*x[j][k][l][m][n][t]* x[0][0][1][m][0][t]+dc*x[j][k][3][m][n][t],0]+

If[(j==0)&&(k==0)&&(l==1)&&(m==1)&&(n==0),-ac*Nf* x[j][k][l][m][n][t]*Sum[x[jj][kk][2][m][1][t],{jj,3,6},{kk,0,3}]+ dc*Sum[x[jj][kk][3][m][1][t],{jj,3,6},{kk,0,3}],0]+

If[(j>2)&&(l==3)&&(m==1)&&(n==1), ac*Nf*x[0][0][1][m][0][t]*x[j][k][2][m][n][t]-dc*x[j][k][l][m][n][t], 0]

**5) Binding and unbinding between CHRONO-PER2 and GSK3b**

x[j][k][l][m][n]'=

If[(j>2)&&((l==0)||(l==1)),-If[m==1,Nf,1]*agp* x[j][k][l][m][n][t]*x[0][0][2][m][0][t]+dg*x[j][k][l+2][m][n][t],0]+

If[(j==0)&&(k==0)&&(l==2)&&(n==0),-If[m==1,Nf,1]*agp* Sum[x[jj][kk][ll][m][nn][t],{jj,3,6},{kk,0,3},{ll,0,1},{nn,0, 1}]*x[j][k][l][m][n][t]+ dg*Sum[ x[jj][kk][ll][m][nn][t],{jj,3,6},{kk,0,3},{ll,2,3},{nn,0,1}], 0]+

If[(j>2)&&((l==2)||(l==3)), If[m==1,Nf,1]*agp*x[j][k][l-2][m][n][t]*x[0][0][2][m][0][t]- dg*x[j][k][l][m][n][t],0]

**6) Binding and unbinding between CHRONO and BMAL1-CLOCK**

x[j][k][l][m][n]'=

If[(j==0)&&(k>0)&&(l==0)&&(m==1)&&(n==0),-cbbin*Nf* x[j][k][l][m][n][t]*x[0][0][0][m][1][t]+uncbbin*x[j][k][l][m][1][t], 0]+

If[(j==0)&&(k==0)&&(l==0)&&(m==1)&&(n==1),-cbbin*Nf* Sum[x[0][kk][0][m][0][t],{kk,1,3}]*x[j][k][l][m][n][t]+ uncbbin*Sum[x[0][kk][0][m][n][t],{kk,1,3}],0]+

If[(j==0)&&(k>0)&&(l==0)&&(m==1)&&(n==1),cbbin*Nf*x[j][k][l][m][0][t]*x[0][0][0][m][n][t]- uncbbin*x[j][k][l][m][n][t],0]

**7) PER2-CHRONO subcellular translocation**

x[j][k][l][m][n]'=

If[((j==2)||(j==4)||(j==5)||(j==6))&&(m==1),-ne* If[(n==0),1,0]*x[j][k][l][m][n][t]+If[(n==0),1,0]*nl*x[j][k][l][0][n][t],0]+

If[((j==2)||(j==4)||(j==5)||(j==6))&&(m==0), ne*If[(n==0),1,0]*x[j][k][l][1][n][t]-If[(n==0),1,0]*nl*x[j][k][l][m][n][t],0]

**8) CHRONO degradation**

x[j][k][l][m][n]'=

If[(j==0)&&(k==3)&&(l==0)&&(n==0),-urt*x[j][k][l][m][n][t], 0]+

If[(j==0)&&(k==3)&&(l==0)&&(m==1)&&(n==1),-urt* x[j][k][l][m][n][t],0]+

If[(j==0)&&(k==0)&&(l==0)&&(m==1)&&(n==1),urt*x[j][3][l][m][n][t], 0]
